# Mammalian Brain Development is Accompanied by a Dramatic Increase in Bipolar DNA Methylation

**DOI:** 10.1038/srep32298

**Published:** 2016-09-02

**Authors:** Ming-an Sun, Zhixiong Sun, Xiaowei Wu, Veena Rajaram, David Keimig, Jessica Lim, Hongxiao Zhu, Hehuang Xie

**Affiliations:** 1 Epigenomics and Computational Biology Lab, Biocomplexity Institute of Virginia Tech, Blacksburg, VA, USA; 2 Department of Biological Sciences, Virginia Tech, Blacksburg, VA, USA; 3 Department of Statistics, Virginia Tech, Blacksburg, VA, USA; 4 Department of Pathology, Children’s Medical center, Dallas 75235 , USA; 5 Department of Pathology, UT Southwestern Medical Center, Dallas, TX 75235, USA; 6 School of Neuroscience, Virginia Tech, Blacksburg, VA, USA; 7 Interdisciplinary Ph.D. Program in Genomics, Bioinformatics, and Computational Biology, Virginia Tech, Blacksburg, VA, USA; 8 Ph.D. Program in Translational Biology, Medicine, and Health, Virginia Tech, Blacksbury, VA 24061, USA

## Abstract

DNA methylation is an epigenetic mechanism critical for tissue development and cell specification. Mammalian brains consist of many different types of cells with assumedly distinct DNA methylation profiles, and thus some genomic loci may demonstrate bipolar DNA methylation pattern, i.e. hypermethylated in one cell subset but hypomethylated in others. Currently, how extensive methylation patterns vary among brain cells is unknown and bipolar methylated genomic loci remain largely unexplored. In this study, we implemented a procedure to infer cell-subset specific methylated (CSM) loci from the methylomes of human and mouse frontal cortices at different developmental stages. With the genome-scale hairpin bisulfite sequencing approach, we demonstrated that the majority of CSM loci predicted likely resulted from the methylation differences among brain cells rather than from asymmetric DNA methylation between DNA double strands. Correlated with enhancer-associated histone modifications, putative CSM loci increased dramatically during early stages of brain development and were enriched for GWAS variants associated with neurological disorder-related diseases/traits. Altogether, this study provides a procedure to identify genomic regions showing methylation differences in a mixed cell population and our results suggest that a set of cis-regulatory elements are primed in early postnatal life whose functions may be compromised in human neurological disorders.

DNA methylation is the most common covalent modification known to occur to mammalian genomic DNA. The importance of DNA methylation has been firmly established for neuronal differentiation, neural plasticity and function throughout the lifespan^1^,^2^,^3^. During early neuronal differentiation, *de novo* DNA methylation occurs at the promoters of germ line-specific genes to repress pluripotency in progenitor cells, while the methylation loss at other promoters activates neuron-specific genes^1^. After birth, neuronal methylation profiles continue to evolve in parallel with developmental plastic changes^4^. In mature brains, substantial DNA methylation changes can result from neuronal activity, for example within hours after electroconvulsive stimulation^5^. The methylation dynamics in neurons have been recognized to be critical for activity-dependent plasticity underlying brain functions including learning and memory^6^,^7^,^8^.

Remarkable heterogeneity in DNA methylation has been observed within mammalian brains, which are comprised of functionally distinct cell subsets^9^. Variations in DNA methylation across brain regions are widespread in human methylomes and consistent within individual brains^9^,^10^. In the mouse brain, unique epigenetic landscapes distinguish different brain regions and account for region-specific functional specialization^11^. To explore cell-subset specific DNA methylation, several studies have been conducted to compare methylation profiles of neuronal and non-neuronal cells using a neuron-specific antibody (NeuN) and fluorescence-activated cell sorting^4^,^12^,^13^,^14^. Compared with those of non-neuronal cells, neuronal methylomes show distinctive DNA methylation signatures with low global DNA methylation and high inter-individual variations^12^. The differentially methylated regions between neuronal and non-neuronal cells are enriched in CpG island shores, enhancers and gene bodies of neuron-specific genes^13^. Despite these advances, current understanding of brain methylation heterogeneity is still very limited and the epigenetic regulatory mechanisms associated with brain cell specification are largely unexplored.

Because the classification of cell types in the brain remains a work in progress^15^, even with the advance of single-cell methylome sequencing technique, the identification of epigenetic marks for each brain cell subset is a daunting task. This prompted us to explore alternative ways to decode the brain methylome derived from unsorted cells. In normal somatic tissues, DNA methylation usually displays a bimodal distribution and the methylation levels between neighboring CpG dinucleotides are strongly correlated^16^. Thus, genomic DNA may be partitioned into two fractions: hypermethylated and hypomethylated^17^. Within heterogeneous tissues, there exist so called cell-subset specific methylated (CSM) loci, which show bipolar methylation patterns. However, bipolar DNA methylation patterns may also result from epigenetic phenomena unrelated to CSM: allele-specific DNA methylation (ASM) and asymmetric DNA methylation. Fortunately, such bipolar methylation loci can be distinguished from the CSM loci. Recently, a mouse ASM map has been generated with brain tissues derived from reciprocal crosses between two distantly related mouse strains^18^. A total of 1,952 CG dinucleotides in 55 discrete genomic loci in the mouse have been identified as imprinted. The number of human imprinted regions were found to be very limited as well^19^ and 51 ASM loci were identified^20^. Additionally, asymmetric DNA methylation may be detected with hairpin bisulfite sequencing technique^21^, which generates methylation data for two complementary DNA strands simultaneously. Thus, the distinction of CSM from other types of bipolar methylation patterns (i.e. ASM and asymmetric DNA methylation) may provide a complementary solution to the cell-sorting-based method to dissect brain epigenetic heterogeneity.

Here, we first developed an analytical procedure to infer CSM loci and applied it to human and mouse brain methylomes^4^. We next used the genome-scale hairpin bisulfite sequencing technique to explore the symmetry of methylation on DNA double strands in human fetal and adolescent brain tissues. We found that, compared with embryonic stem cells^21^, brains exhibit exceedingly higher levels of symmetrical DNA methylation. We further explored the functional relevance of the predicted brain CSM loci *via* integrative “omics” analysis with disease/trait-associated genetic variants and ChIP-seq data for histone modifications. The integrative analysis suggested that putative brain CSM loci are critical elements which may be associated with epigenetic aberrations in human brain diseases.

## Results

### CSM dynamics during mammalian brain development

**Figure 1:**
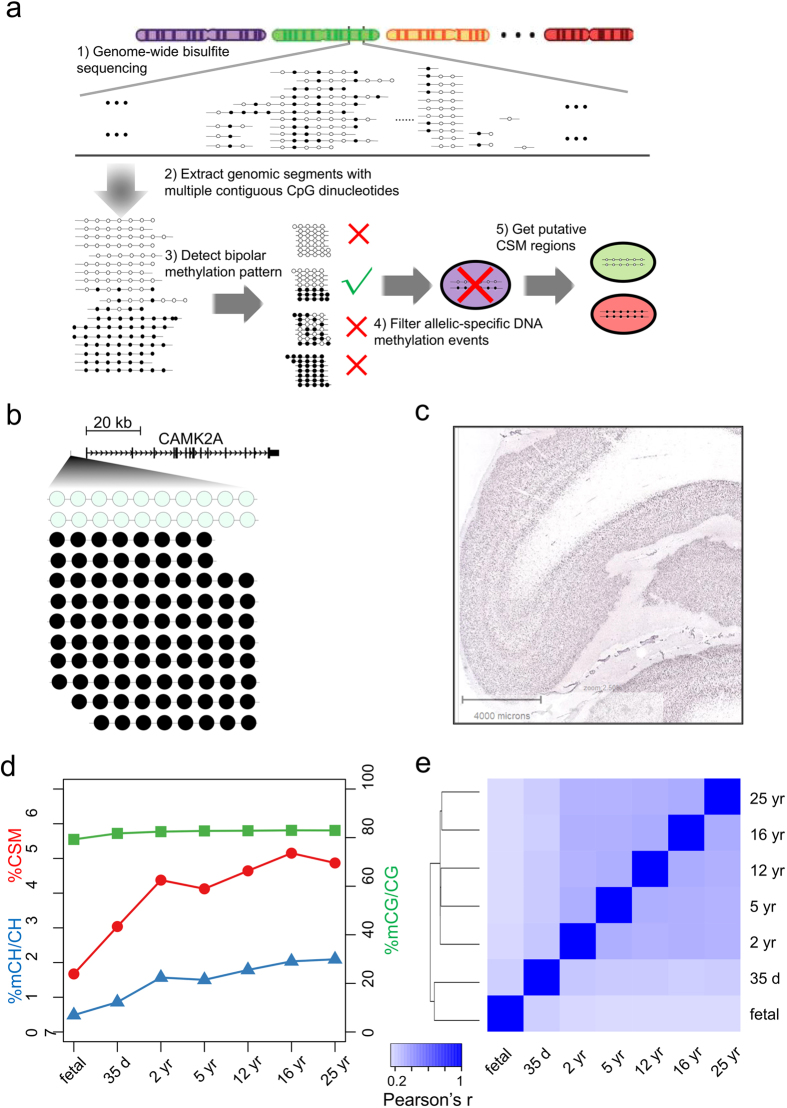
Identification of pCSM loci in human brain methylomes. (**a**) Workflow for the computational inference of pCSM loci. (**b**) Methylation pattern of a predicted CSM loci (Chr5:149675541-149675604) at 6,138 bp upstream of CAMK2A gene in 25 yr brain methylome. (**c**) *In situ hybridization* for CAMK2A in human prefrontal cortex showing positive staining in the excitatory neurons in layers II-VI with absent staining in the glial cells (modified from Allen Brain Atlas; http://human.brain-map.org/ish/specimen/show/80936541?gene=811). (**d**) Changes of mC level in CG and CH context and the percentage of CSM segments after down-sampling normalization during human brain development. (**e**) Hierarchical clustering based on Pearson’s correlations of pCSM statuses predicted for 4-CpG segments in different human brain methylomes.

To investigate brain CSM, we designed a reverse engineering approach to analyze DNA methylation patterns embedded in bisulfite sequencing reads (Fig. 1a). During this procedure, we first excluded potential PCR duplicates and progressively scanned each sequence to extract methylation patterns for genomic segments containing four neighboring CpG dinucleotides. The 4-CpG segments mapped to the same locus were clustered together, and the clusters with at least 10Xs read coverage were selected for further analysis. To increase the certainty of bipolar detection, each selected cluster was required to include at least one completely methylated and one completely unmethylated read concurrently. We further analyzed the distributions of methylation patterns for each cluster to identify bipolar methylated ones using nonparametric Bayesian clustering approach^22^.

For both human and mouse, we re-analyzed eleven methylomes^4^ derived from the frontal cortices from seven developmental stages along with two pairs of fluorescence activated cell sorted NeuN+ and NeuN- cells. After the filtering of imprinted loci, we inferred the rest of bipolar methylated segments as putative cell-subset specific methylated loci, which were denoted as pCSM loci. We identified 5,636 to 96,033 pCSM loci in human autosomes and 3,343 to 57,001 pCSM loci in mouse autosomes (Supplementary Table 1). A recent comparison of neuronal and stem-cell methylomes revealed that cell-subset-specific regulation is associated with a set of low-methylated regions with an average methylation level of 30%^23^. In contrast, we found that the majority of pCSM loci were hypermethylated. For these pCSM loci, completely unmethylated patterns may exist in a small percentage of cells within bulk brain tissues. For instance, a number of bipolar methylated loci were adjacent to the transcription start sites (TSSs) of genes expressed in a specific cell type, such as CAMK2A, which is known to be selectively expressed in excitatory neurons^24^ (Fig. 1b,c).

We next determined the frequency of pCSM for each methylome, which was defined as the percentage of pCSM segments predicted from all 4-CpG segments with at least 10Xs read coverage. The pCSM frequencies ranged from 1.2% in human fetal frontal cortex to 4.6% in 55-year-old NeuN+ cells. To enable a quantitative comparison across developmental stages, we focused on the 4-CpG segments with at least 10Xs read coverage in all stages (n = 171,178 for human; n = 35,605 for mouse). Since the number of pCSM loci identified from methylomes highly depends on the sequencing depth, we performed down-sampling normalization to minimize the bias that results from uneven sequencing depth. As previously reported^4^, the levels of global DNA methylation remain constant during brain development (Fig. 1d). Interestingly, we observed a dramatic increase in pCSM frequency during early stages of brain development in both human (Fig. 1d) and mouse (Supplementary Fig. 2). The pCSM frequency was 1.7% for human fetal frontal cortex, increasing to 4.4% in two-year-old, and remained stable at later stages. This result suggests that a growing number of genomic loci are involved in brain cell specification at early stages and reach a plateau in adult brains.

We further performed pair-wise comparisons of pCSM profiles and determined their correlations among developmental stages. In coherence with the swiftly established CSM status during early developmental stages, the fetal and 35-day brains were clustered together and maturing brains gave rise to another cluster (Fig. 1e). For human and mouse brains, the closer the two developmental stages are, the higher the correlation in pCSM profiles (Fig. 1e, Supplementary Fig. 2b and Supplementary Table 2). Compared with non-neuronal cells, both human and mouse neurons exhibited higher frequencies of pCSM (Supplementary Fig. 3a,b), and the pCSM profiles were conserved for the same cell types (Supplementary Fig. 3c,d). This suggests that more genomic loci are needed to encode highly diverse neuronal cells than non-neuronal cells. Such a high epigenetic heterogeneity within NeuN+ cell population may explain the higher inter-individual methylation variations observed for NeuN+ cells in a recent study^12^. In addition, for both human and mouse brain methylomes, the percentages of pCSMs shared within a cell type are consistently higher than those between different cell types (Supplementary Table 3 and Supplementary Fig. 4).

It has been reported that the levels of non-CpG methylation (mCH) and 5-hydroxymethylcytosine (5hmC) increase during neuronal development^4^,^25^,^26^,^27^. This prompted us to investigate whether they were related to the establishment of CSM loci. Similar to the previous report^4^, an increase in the mCH frequency was observed during brain development, while the mCG levels remained relatively stable (Fig. 1d and Supplementary Fig. 2a). We next examined the levels of mCH within pCSM loci and controls (all 4-CpG segments with at least 10Xs coverage but with CSM loci excluded) in fetal and adult brains, NeuN+ and NeuN- cells (Supplementary Fig. 5). For both CSM and control 4-CpG segments, the level of mCH was positively correlated with the level of mCG (Pearson’s *r* ranges from 0.05 to 0.41) in all samples examined. We further compared pCSM segments with the controls at the same interval of mCG level and found that pCSM loci tend to have a higher level of mCH, especially in adult brain and neurons. In addition, we observed that hmC was also enriched in pCSM loci (Supplementary Fig. 6). This suggested that the establishment of pCSM loci may have mechanistic links with the increasing 5hmC and mCH levels during neuronal development.

### Human frontal cortex exhibits high methylation fidelity

As mentioned previously, the predicted brain CSM loci may result from the methylation difference within a DNA molecule, i.e. asymmetric DNA methylation. To assess the levels of asymmetric DNA methylation in the brain, we performed genome-scale hairpin bisulfite sequencing with gray and white matters from human fetal and adolescent frontal cortices (Supplementary Table 4). For all samples, bisulfite conversion rates were determined to be over 99.7% with the spike-in lambda DNA control. Despite the variation in the numbers of sequence reads generated for these samples, we found that the genome-wide average of methylation level and fidelity could be denoted with data from as low as 10^4^ randomly generated read pairs (Supplementary Fig. 7). All four brain tissues demonstrated exceedingly high methylation fidelity, which was defined as the percentage of CpG dyads with symmetrical methylation patterns. We recently determined that in undifferentiated and differentiating mouse embryonic stem cells (ESCs), the average methylation fidelities were 88.5% and 91.9% respectively^21^. Here we found that in the human fetal brain, the average methylation fidelities were 94.9% and 95.0% for cortical grey and white matters, respectively. These numbers increase to 95.6% for grey matter and 95.8% for white matter in the adolescent brain (Supplementary Table 4).

**Figure 2:**
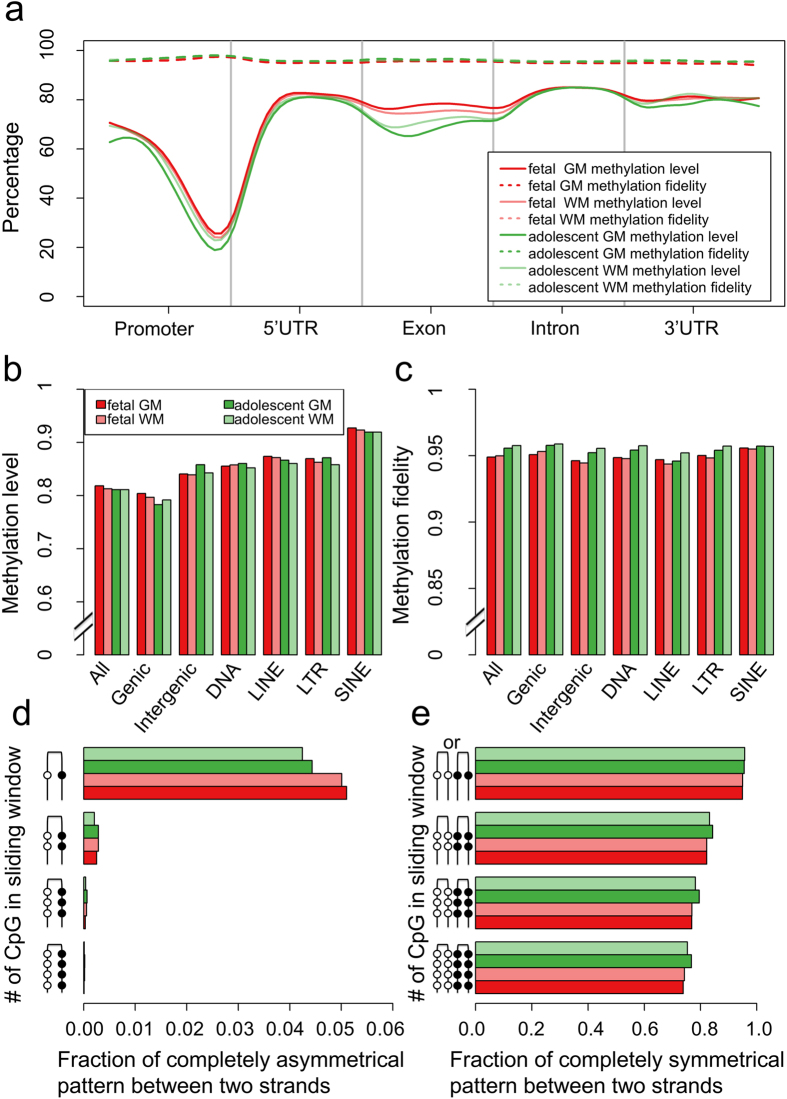
Hairpin bisulfite sequencing for human fetal and adolescent brains. (**a**) Methylation level and fidelity along different gene-associated regions. Each genomic region was divided into 20 equal-sized bins. The smoothed lines represent the mean methylation level (solid lines) and methylation fidelity (dashed lines). (**b**) Methylation level in different genomic regions. (**c**) Methylation fidelity in different genomic regions. (**d**) Fraction of bipolar methylation pattern using different sliding windows. (**e**) Fraction of symmetrical (un)methylation pattern using different sliding windows. GM and WM represent gray matter and white matter, respectively.

Next we examined the genomic distribution of asymmetrically methylated CpG dyads. Similar to previous observation made with mouse ESCs^21^, we found that the average methylation levels decrease to approximately 20% but the average methylation fidelity increase slightly approaching the TSSs (Fig. 2a). By comparing the methylation fidelity for CpG dyads within +/−1 kb from TSS against all other CpG dyads, we found that CpG dyads near TSSs could show small (~2%) yet significant increase of methylation fidelity (p-value between 0.02 and p < 2.2e-16 for different samples; Wilcoxon Rank Sum Test). The methylation levels of exons showed high variance across samples and were lower than those of introns from the same samples. However, the methylation fidelity remained constantly high along the entire gene body in all samples. Furthermore, compared with genic regions, intergenic regions and repetitive elements (in particular SINE) have 3–10% higher methylation levels (Fig. 2b) and their methylation fidelities were above 94% in all four brain samples (Fig. 2c). We next questioned whether asymmetrically methylated CpG dyads tend to cluster together and thus result in bipolar DNA methylation patterns. We applied sliding windows with one to four adjacent CpG dyads to all sequence reads and compared the methylation patterns between two complementary DNA strands. DNA methylation pattern, herein, is defined as the combination of methylation statuses of adjacent CpG dinucleotides on the same DNA strand. We focused on CpG dinucleotides showing the same methylation pattern (all methylated or all unmethylated) in a given sliding window. In all four brain samples, the frequency of asymmetrically methylated CpG dyads decreased more than 200 times from approximately 5% for a single CpG dyad to 0.02% for a sliding window of four CpG dyads (Fig. 2d). In contrast, the frequency of symmetrical methylation patterns decreased from approximately 95% for a single CpG dyad to 74% for a sliding window of four CpG dyads (Fig. 2e). Thus, in both fetal and adolescent brain methylomes, genomic segments with four neighboring CpG dinucleotides showing completely methylated or unmethylated patterns are more likely to have symmetrical (74%) rather than asymmetrical (0.02%) methylation patterns. Therefore, high methylation fidelity observed in the brain indicated that the predicted CSM loci result from methylation differences among brain cells instead of asymmetric DNA methylation.

### CSM regions are enriched in non-CGI promoters and enhancers

**Figure 3:**
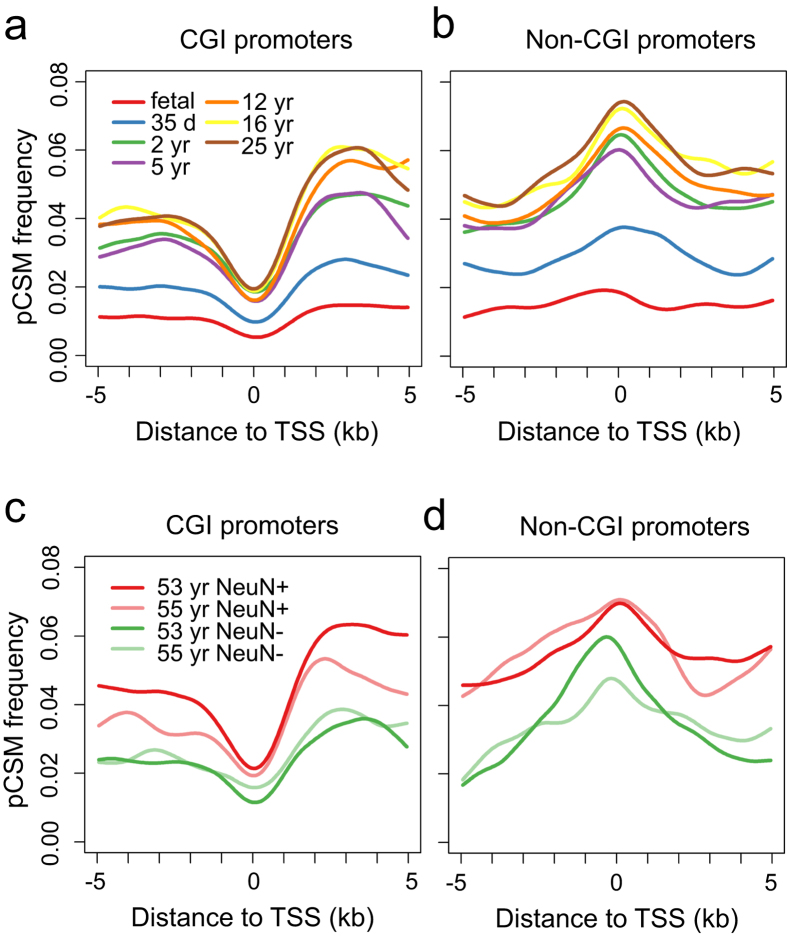
Aggregate plots of pCSM frequency surrounding TSSs. pCSM frequency was calculated as the percentage of 4-CpG segments predicted to be pCSM. pCSM segments are depleted from CGI promoters (**a,c**), while over-represented in non-CGI promoters (**b,d**).

To evaluate the functional relevance of pCSM segments, we examined the occurrence of pCSM loci in promoters and identified their associated genes. Based on the presence of CpG islands (CGIs), gene promoters may be classified into CGI promoters and non-CGI promoters. Most CGI promoters are associated with housekeeping genes, while non-CGI promoters are usually for genes of non-ubiquitous (tissue or cell-subset specific) expression^28^. We observed that pCSM regions were depleted from CGI promoters but enriched in non-CGI promoters (Fig. 3). This finding is in agreement with previous studies, which reported that differentially methylated regions between human neuronal and glial cells are depleted from CGI-promoters^13^.

Various histone modifications have been used to generate genome-wide maps of chromatin state and to annotate regulatory elements^29^. Active histone marks such as H3K4me3 and H3K9ac are signatures of active promoters, while H3K4me1 marks enhancers in active or poised state and H3K27ac identifies active enhancers^30^. Based on the chromatin maps including H3K4me1, H3K4me3 and H3K27ac modifications in the adult human brain^31^, we annotated genomic regions for active promoters, poised enhancers and active enhancers. Subsequently, the pCSM occurrence surrounding these regulatory elements was examined. We found that pCSM loci were overrepresented in both active non-CGI promoters and active/poised enhancers (Supplementary Fig. 8). In contrast, no significant change on pCSM frequency was observed in surrounding genomic regions enriched for several other histone marks^31^ including H3K9ac, H3K9me3, H3K27me3 and H3K36me3. This is in consistent with the fact that the enhancers are usually highly cell-subset specific^29^,^30^.

### CSM regions are associated with brain functions and rich in brain disease/trait-associated SNPs

**Figure 4:**
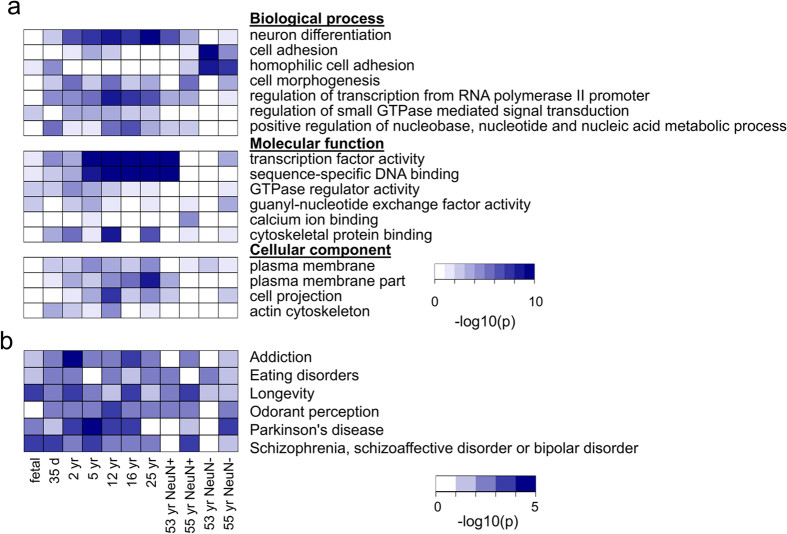
Functional annotation analyses for pCSM loci. (**a**) GO enrichment analysis for genes associated with pCSM loci during human brain development and cell specification. Genes associated with pCSM were defined as those with pCSM loci within 10 kb from their TSSs. The color represents p-values after Bonferroni correction. The GO terms were grouped as “biological process”, “molecular function” and “cellular component” (for the full result, see Supplementary Table 6). (**b**) Diseases/traits associated with pCSM loci. The color represents p-values determined by permutation tests with 10,000 iterations.

For genes with pCSM loci within 10 kb of their TSSs, we performed GO enrichment analysis using DAVID functional annotation tools^32^. Not surprisingly, we found that these genes were involved in functions including neuronal differentiation, cell morphogenesis, transcription factor activity and cell projection (Fig. 4a and Supplementary Table 5). This result indicates that the identified pCSM segments may play important roles in the epigenetic regulation of brain cell specification and morphogenesis. Interestingly, remarkable differences in GO terms enriched were observed among different developmental stages and between cell types. GO terms including “neuron differentiation” and “cell morphogenesis” were enriched for NeuN+ cells, while “hemophilic cell adhesion” and “plasma membrane” were enriched for NeuN- cells. In addition, GO terms such as “neuron differentiation” and “transcription regulation” were highly enriched in maturing brains, but not in fetal brains. These results imply that the pCSM profiles in brains are functionally important for brain development and cell-subset specificity.

To explore whether the identified pCSM regions can provide insight into SNP variants associated with disease phenotypes, we performed statistical analysis on the overlap between disease/trait-associated SNP variants and pCSM regions. With the disease/trait-associated SNPs documented in NHGRI genome-wide association studies (GWAS) catalog^33^ and their proxy SNPs^34^ in strong linkage disequilibrium, we observed significant enrichment of SNP variants associated with major brain diseases/traits in the pCSM regions, including odorant perception, Schizophrenia, addiction, eating disorders, and Parkinson’s disease (Fig. 4b). The SNPs associated with these diseases/traits were recurrently enriched in pCSM regions identified in the eleven human methylomes. These results not only suggest functional importance of the pCSM regions but also provide a basis for better understanding the underlying epigenetic mechanism of the ‘common disease-common variant’ association. Further studies are needed to determine whether the brain disease/trait-associated SNPs correlate with methylation alterations at pCSM regions within specific cell-subsets and at specific developmental stages.

## Discussion

The high degree of cellular complexity within the human brain has been well recognized. However, little is known of its epigenetic heterogeneity. To our knowledge, this study is the first attempt to systematically exploit the dynamics of epigenetic heterogeneity associated with mammalian brain development. The ideal approach to identify cell-subset specific methylation is to determine the methylomes of single cells. Recently, three labs^35^,^36^,^37^ have generated methylomes at the single-cell level for embryonic stem cells. Unfortunately, the genome coverage of single-cell methylome data is frequently lower than 5%. This greatly limits the comparison of methylomes derived from different cells. Notably, single-cell methylation data alone cannot rule out methylation variations within a cell, i.e. allelic-specific methylation and asymmetric DNA methylation.

With hairpin bisulfite sequencing technique, we were able to estimate the contribution of asymmetric DNA methylation to bipolar methylation patterns observed in methylomes derived from brain tissues. We observed that human fetal and adolescent brain methylomes are with high methylation fidelity. This probably results from the fact that DNA methyltransferases in the brain postmitotic cells have sufficient time to faithfully replicate parental DNA methylation patterns. Such high methylation fidelity in brain tissues leads to an extremely low frequency of asymmetrical DNA methylation. In both fetal and adolescent brain methylomes, as low as 0.02% of genomic segments with four neighboring CpG dinucleotides showing completely methylated or unmethylated patterns are with asymmetrical DNA methylation patterns. Currently, the catalogue of brain cell types is under debate^15^ and it remains impossible to clearly define every cell subtype in the brain with existing methods. However, the high methylation fidelity of brain methylomes observed in this study indicates that assessing large populations of methylation patterns might aid in this endeavor. In light of this finding, we developed a unique approach to dissect brain methylomes and identified genomic loci potentially associated with brain cell specification. Such a tool provides an alternative and complementary solution to the cell-sorting-based approach to dissect brain epigenetic heterogeneity. There are several limitations in our study. Due to the still prohibitive cost of sequencing, most current methylomes were generated at around 10Xs coverage. Considering the numerous cell types in mammalian brains, we are aware that this study may not capture all the methylation variations among brain cells. Recently, forty-nine monoallelic methylated loci have been identified in genic regions^38^. Although the majority of bipolar methylated loci in brain are unlikely caused by random monoallelic methylation, we cannot completely rule out such a possibility. For instance, the mosaic methylation patterns at the promoters of protocadherin-α gene cluster may help the monoallelic and combinatorial expression of variable isoforms in individual Purkinje cells^39^. In addition, our analytical procedure may be applied to regular bisulfite sequencing data for other normal tissues, but not for tumors or fast-dividing cells with low methylation fidelity, in which prevalent asymmetric DNA methylation may contribute substantially to bipolar DNA methylation^21^.

In this study, we observed a dramatic increase in the frequency of pCSM loci during early postnatal brain development. This intriguing result indicates that early postnatal stages are critical for mammalian brain development to create the diversity at the epigenetic level although neurogenesis has largely completed at birth. During the first few years for human and weeks for mouse, we found that brain cells gain significant variations in DNA methylation patterns. The established pCSM loci were highly enriched in regulatory elements controlling gene expression, i.e., enhancers and non-CGI promoters. In addition, the functional annotation analyses indicate that brain pCSM loci are associated with genes relevant to brain development and neuron-specific functions. By exploring the NHGRI GWAS catalog, we further observed that the strong links between brain pCSM loci and the genomic loci associated with human neurological disorders, including addiction, Parkinson’s diseases, schizophrenia, etc. Thus, the brain pCSM loci predicted and the tool we provided in this study could inform the candidate genomic loci associated with epigenetic aberrations in neurological disorders.

Lastly, the pCSM loci identified in this study may serve as epigenetic markers for specific cell lineages and associated gene networks possibly involved in epigenetic regulatory processes determining cell fate. During cellular differentiation, establishment of cell-subset specific methylation patterns enables cells with same genetic composition to stably silence specified genes and exhibit distinct phenotypes^40^. The “on/off” combinations of epigenetic switches may be used to classify cells within the frontal cortex into distinct subsets. Such information on brain developmental trajectory will help design a probe set for monitoring developmental stages of specific cells, which will be useful in pre-clinical models including neural stem cell differentiation and/or reprogramming. The information about cell-subset specific methylated loci may be integrated with gene expression profile, morphologic, neurochemical and electrophysiological properties of specific cell-subsets. Such efforts will extend our understanding of brain cell identity, and may enable reprogramming strategies for specific neuronal cell types with parameters at the epigenetic level for standardized quantification.

## Methods

### Accession codes

The hairpin bisulfite sequencing data generated in this study, including relevant processed data files, have been deposited in NCBI Gene Expression Ominibus (GEO) under accession number GSE70982. Additional data used in this manuscript were summarized in Supplementary Table 6.

### Collection and dissection of post-mortem human brain tissues

This study involves pre-existing de-identified human specimens (IRB-exempt research), and thus an ethics approval is not required and no patient consent is needed. The study was carried out in accordance with the approved guidelines at Virginia Tech and UT Southwestern. De-identified postmortem human brain tissues were acquired from autopsies performed at Children’s Medical Center, University of Texas Southwestern, Dallas. Gray and white matters from frontal cortices were obtained from a fetal and an adolescent, who died of non-neurological diseases. Hematoxylin and eosin stained sections of formalin fixed paraffin embedded (FFPE) brain tissue from standard sections of the left frontal cortex (watershed area, adjacent to the frontal horn of the lateral ventricle) were reviewed. Representative cortical gray matter and white matter areas were demarcated and their corresponding areas were mapped on the tissue block. Three to six 1 mm tissue cores were taken from these areas and labeled as cortical gray matter or white matter. For each tissue sample, deparaffinization was performed using 100% xylene with gentle shaking at room temperature (RT) for 15 min, repeated twice and then subjected to 100% ethanol wash, 3 times. The sections were dried on bench for 1 h, and then 1 ml lysis buffer (50 mM Tris, 25 mM EDTA, 100 mM NaCl, 0.5% Tween-20/SDS, pH 8.0) was added with 40 ul proteinase K (Ambion). After gently shaking overnight at 55 °C, heat-inactivation at 90 °C for 1 h was performed and samples were cooled down gradually, RNA digestion was performed using 5 ul RNase cocktail (Ambion) at 37 °C for 2 h. Finally genomic DNA was isolated with phenol/chloroform extraction followed by ethanol precipitation.

### Genome-scale hairpin bisulfite sequencing

Hairpin bisulfite-seq library construction was performed according to previously described protocol^21^ with slight modifications. Briefly, 10 ug genomic DNA of each sample was spiked with 0.02% unmethylated Lambda DNA (Promega) and sonicated to 200 bp fragments with Covaris. After MseI and MluCI digestion (NEB), end repair and dA tailing, genomic DNA fragments were ligated to Biotin-modified hairpin adapter (5′P-GGCCAGCTGCA AG/iBiodT/GAAGCAGCTGGCCT-3′, IDT). After captured with Dynabeads^®^ MyOne™ Streptavidin C1 beads (Invitrogen), genomic DNA fragments were subjected to bisulfite conversion using the EpiTect Bisulphite Kit (Qiagen), PCR, and pair-end sequenced using Illumina MiSeq and HiSeq 2000. Illumina Sequencing services were performed at the genomic core of Virginia Bioinformatics Institute.

### Hairpin bisulfite sequencing data analysis

The hairpin bisulfite sequencing reads were processed and aligned similar to a previous study^21^. For each read, adaptor and hairpin sequences were searched with cross_match. Additional searches on the 3′-end of sequence reads were conducted to eliminate any sub-string derived from a hairpin sequence adaptor. Then, the processed read pairs were globally aligned using Needleman-Wunsch algorithm. After trimming the overhangs of the aligned sequences, original sequences were recovered for read pairs with at least 90% identity between two arms. Finally, the recovered original sequences were mapped to human reference (hg19) using Bowtie 2^41^ with parameters (-N 1 –L 22), and only uniquely mapped reads were retained. Methylation level and fidelity were calculated according to a previous study^21^. Statistics including the number of total uniquely mapped reads, genome coverage, CpG coverage, and the average sequencing depth were summarized in Supplementary Table 4.

### MethylC-Seq data analysis to infer brain pCSM loci

MethylC-Seq data for human and mouse frontal cortex^4^ were retrieved from NCBI Sequence Read Archive (SRA) with accession SRP026048. Due to low sequencing depth, several samples (Hs 55yr tissue, Hs 64yr, Mm 6wk NeuN+ and Mm 6wk NeuN-) were excluded from pCSM prediction. Read processing was performed as previously described^4^. The processed reads were aligned to the corresponding human (hg19) or mouse (mm10) reference genomes using Bismark^42^ with parameters −n 2 −l 50. After PCR duplicates were removed, methylation callings were extracted. Basic statistics can be found in Supplementary Table 1.

To normalize sequencing depth across methylomes, a “down-sampling” strategy was applied at each 4-CpG segment. For the methylomes analyzed, we first obtained a set of common 4-CpG segments, which were covered by at least ten reads in all samples. For each 4-CpG segment, we determined the minimum sequencing depth *D*_min_ in these samples. Then, for a given sample, if its sequencing depth was bigger than *D*_*min*_, reads were randomly discarded from the corresponding data set until reaching *D*_*min*_. For each sample, the “down-sampling” procedure was repeated 100 times for each segment, and the possibility to detect CSM pattern was calculated. Such “down-sampling” strategy was adopted to compare brain methylomes classified into the following four groups: human developmental stages, human cell types (NeuN+ vs NeuN-), mouse developmental stages, and mouse cell types (NeuN+ vs NeuN−).

Nonparametric Bayesian clustering^22^ was used to identify the 4-CpG segments with biplolar methylation patterns. We first scanned all possible segments with four neighboring CpGs within a sequence read. The 4-CpG segments covered with at least ten reads were used for pCSM prediction. CSM segments in chromosome X, Y and known imprinted regions for mouse^18^ and human^20^ were excluded from further analysis. The overlapped pCSM segments were further merged to pCSM regions or so-called pCSM loci.

### Genome annotation and gene ontology analysis

The annotations for genomic regions, including transcripts and CpG islands, were downloaded from UCSC genome browser^43^. Promoters were arbitrarily defined as regions 2 kb upstream of each TSS. Promoters were further classified as CGI and non-CGI groups based on whether they are overlapped with CGIs. pCSM associated genes were defined as those with at least one pCSM segment identified within 10 kb from their TSSs. The background gene list was determined for each sample to include genes with at least one 4-CpG segment within 10 kb from their TSSs. GO enrichment analysis was performed using DAVID functional annotation tools^32^.

### Integrative “Omic” data analysis

ChIP-seq data for histone modifications (Supplementary Table 6) were collected from previous publications. The histone modifications include H3K4me1, H3K4me3, and H3K27ac for mouse brain^44^, and H3K4me1, H3K4me3, H3K9ac, H3K9me3, H3K27ac, H3K27me3, and H3K36me3 for human brain^31^. The coordinates of histone peaks were converted to mm10 by using UCSC liftOver. Based on histone modifications including H3K4me1, H3K4me3, and H3K27ac, several types of important regulatory elements could be annotated^29^,^45^. First, all overlapped peaks for these three histone modifications were merged to form the merged-peak regions. By examining the histone modification occupancy of each merged-peak region, regulatory elements were annotated as: 1) Active promoters: with H3K4me3. They were further classified into two groups based on their overlapping status with CGIs. 2) Active enhancers: with H3K27ac but without H3K4me3. 3) Poised enhancers: with H3K4me1 but without H3K27ac or H3K4me3. The microarray and ISH data for human brain used to validate our result were adopted from Allen Brain Atlas^46^,^47^.

GWAS variants information was collected from the NHGRI GWAS catalog (http://www.genome.gov/GWAStudies, downloaded on Sept 17, 2014). For each disease/trait, its associated SNPs were collapsed into one unique set, then extended by propagating disease/trait associations to proxy SNPs using the SNAP search tool^34^ based on linkage disequilibrium (r^2^ > 0.8) between SNPs (within 250 kb) in any of the three populations in the 1000 genomes project pilot data^48^. With this approach, the total 15,578 SNP-disease associations lead to a set of 379,762 proxy SNPs corresponding to 1,127 diseases/traits. Next, for each of the eleven human brain methylomes, locations of the identified pCSM regions were obtained after a 100 bp extension on both up and down-streams. Enrichment analysis of GWAS variants in pCSM regions for each disease/trait was done by testing whether the overlapping frequency of the disease-associated SNPs was significantly higher than expected. To calculate enrichment p-values, a permutation test was performed by randomly shuffling (shifting along the circulated genome) the locations of pCSM regions for 10,000 iterations. The number of SNPs together with their proxy SNPs within 100 bp from pCSM regions was counted in each iteration to generate a null distribution. Finally, the total 1,127 diseases/traits were ranked in descending order by counting their significant overlapping p-values shown in the eleven human methylomes. This recurrent enrichment in multiple methylomes provides strong evidence for the functional importance of pCSM regions.

## Additional Information

**How to cite this article**: Sun, M.-a. *et al*. Mammalian Brain Development is Accompanied by a Dramatic Increase in Bipolar DNA Methylation. *Sci. Rep.* **6**, 32298; doi: 10.1038/srep32298 (2016).

## Supplementary Material

Supplementary Information
