# Supplementary material for: Mammalian Brain Development is Accompanied by a Dramatic Increase in Bipolar DNA Methylation
Source: Sci Rep. 2016 Sep 2;6:32298. doi: 10.1038/srep32298 (PMC5010034; doi:10.1038/srep32298)
Supplement: Supplementary Information [file srep32298-s1.pdf]

Supplementary figures and tables for:

Mammalian Brain Development is Accompanied by a Dramatic Increase in Bipolar DNA Methylation

Ming-an Sun[1,9], Zhixiong Sun[1,2,9], Xiaowei Wu[3], Veena Rajaram[4,5], David Keimig[1], Jessica Lim[1,2], Hongxiao Zhu[3], Hehuang Xie[1,2, 6,7,8]

[1]Epigenomics and Computational Biology Lab, Biocomplexity Institute of Virginia Tech; [2]Department of Biological Sciences, [3]Department of Statistics, [6]School of Neuroscience, [7]Interdisciplinary Ph.D. Program in Genomics, Bioinformatics, and Computational Biology, [8] Ph.D. Program in Translational Biology, Medicine, and Health, Virginia Tech, VA 24061; [4]Department of Pathology, Children's Medical center, [5]Department of Pathology, UT Southwestern Medical Center, Dallas, TX 75235; USA

[9]These authors contributed equally to this work.

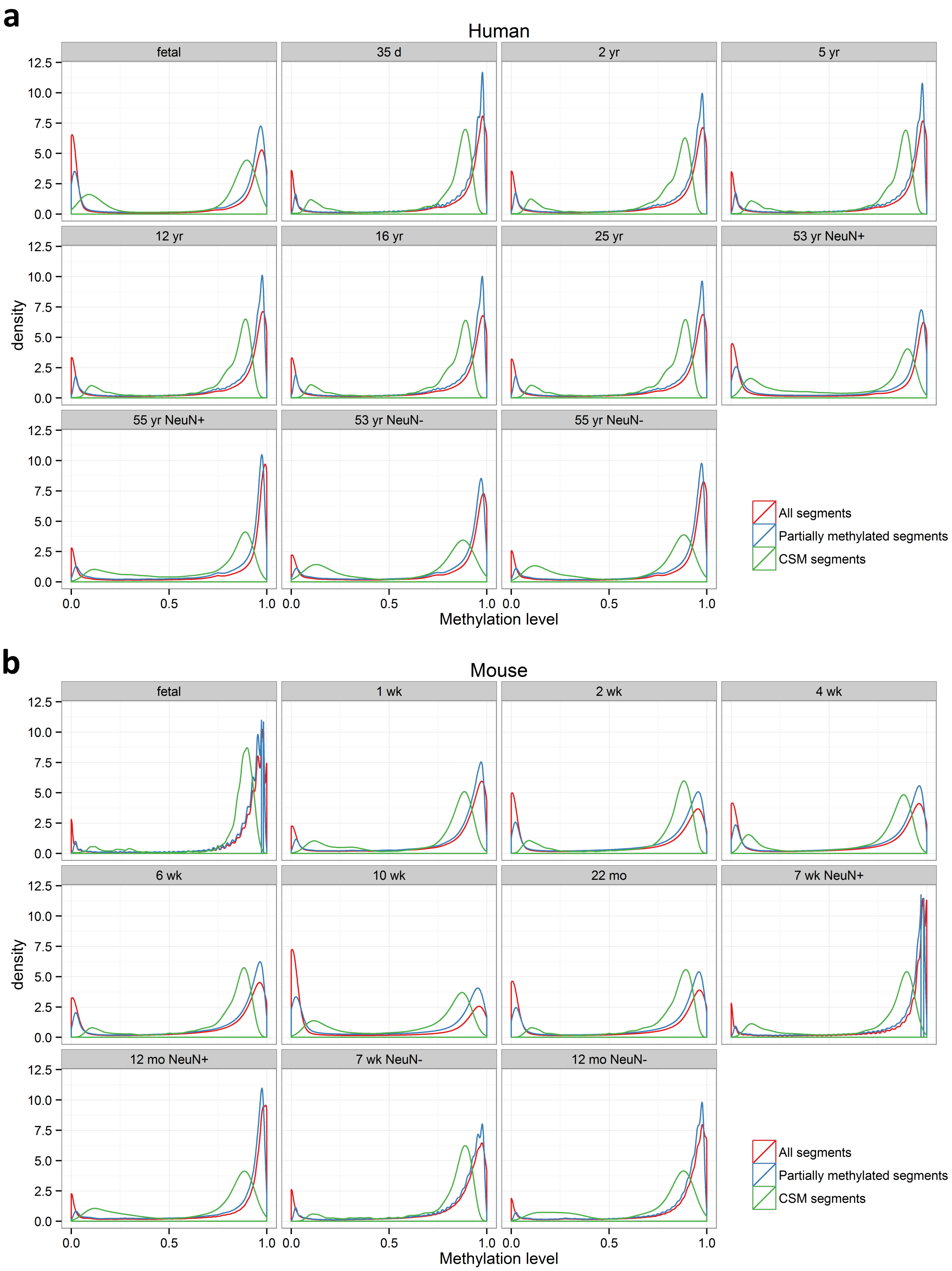

Supplementary Figure 1 (Xie)

The methylation level distribution for 4-CpG segments in human (a) and mouse (b)

For each methylome, the distributions of methylation levels were shown for all 4-CpG segments, partially methylated 4-CpG segments and pCSM 4-CpG segments.

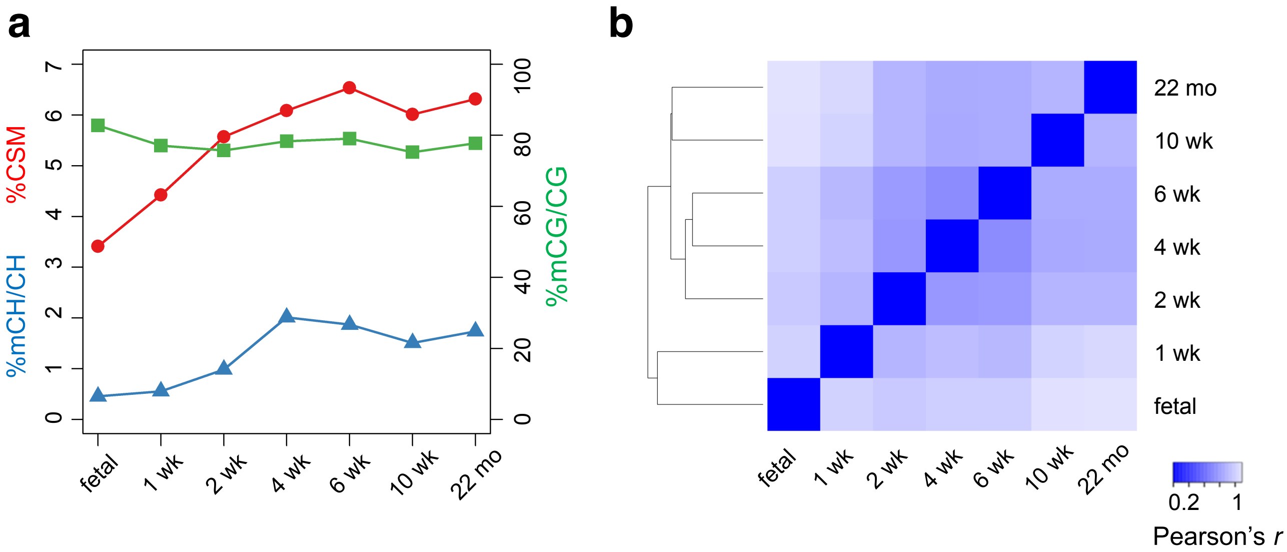

Supplementary Figure 2 (Xie)

Dynamic of CSM during mouse brain development

(a) Changes of mC levels in CG and CH context and the percentage of pCSM segments during mouse brain development. **(b)** Hierarchical clustering based on Pearson's correlations of pCSM statuses predicted for all 4-CpG segments in different mouse brain methylomes.

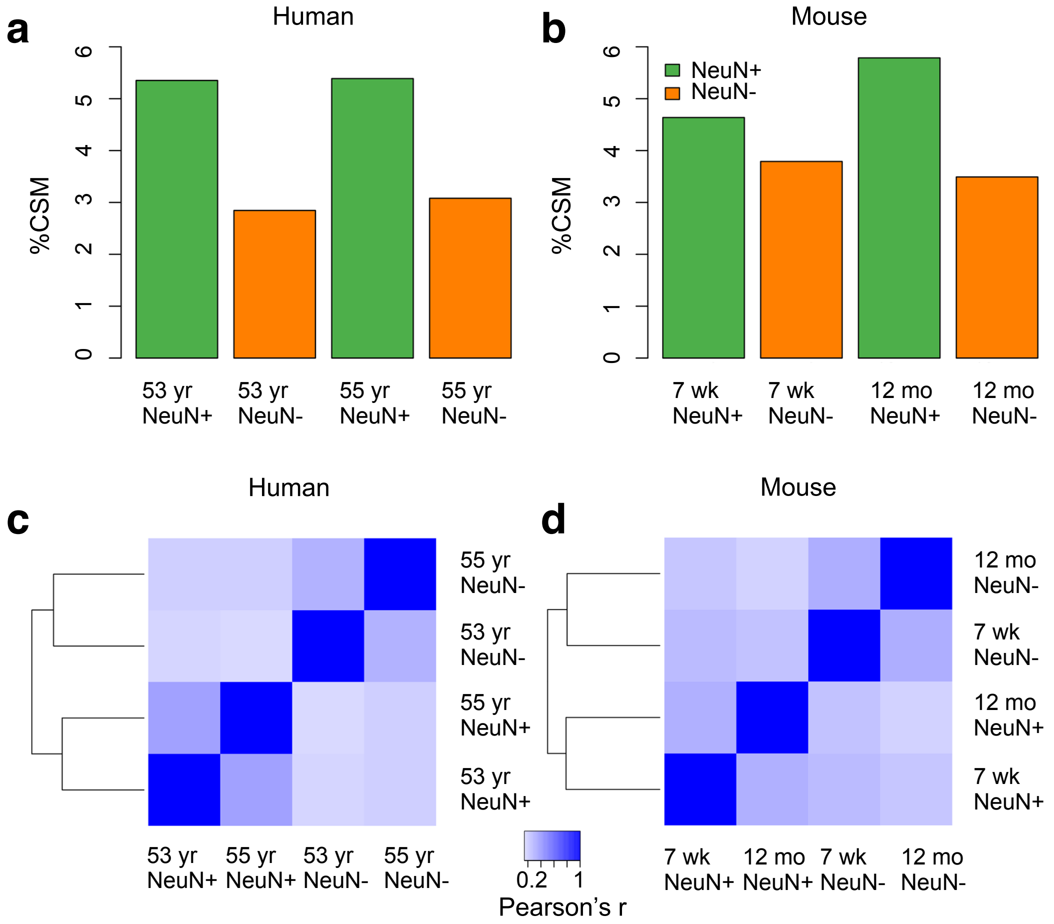

Supplementary Figure 3 (Xie)

Global pCSM profiles in NeuN+ and NeuN- cells

(a,b) The percentage of pCSM 4-CpG segment in the methylomes of NeuN+ and NeuN- cells for human and mouse, respectively. **(c,d)** Hierarchical clustering based on Pearson's correlations of pCSM statuses predicted for all 4-CpG segments in the methylomes of NeuN+ and NeuN- cells for human and mouse, respectively.

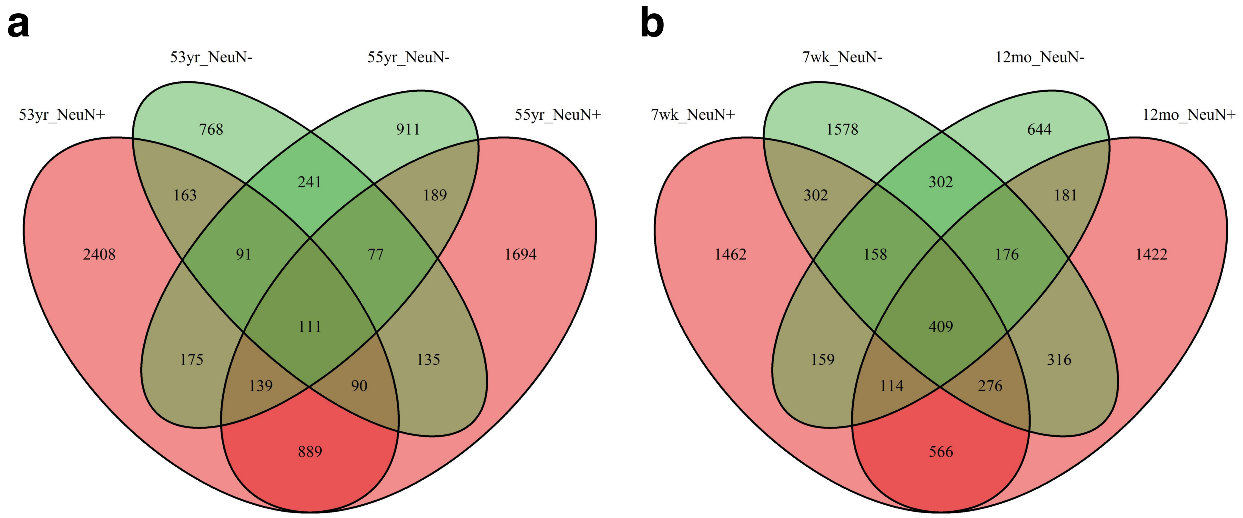

Supplementary Figure 4 (Xie)

Venn diagrams show the overlapping of pCSM segments between the NeuN+ and NeuN- cells

The figures show the overlapping of pCSM segments between the purified NeuN+ and NeuN- samples for human (**a**) and mouse (**b**), respectively.

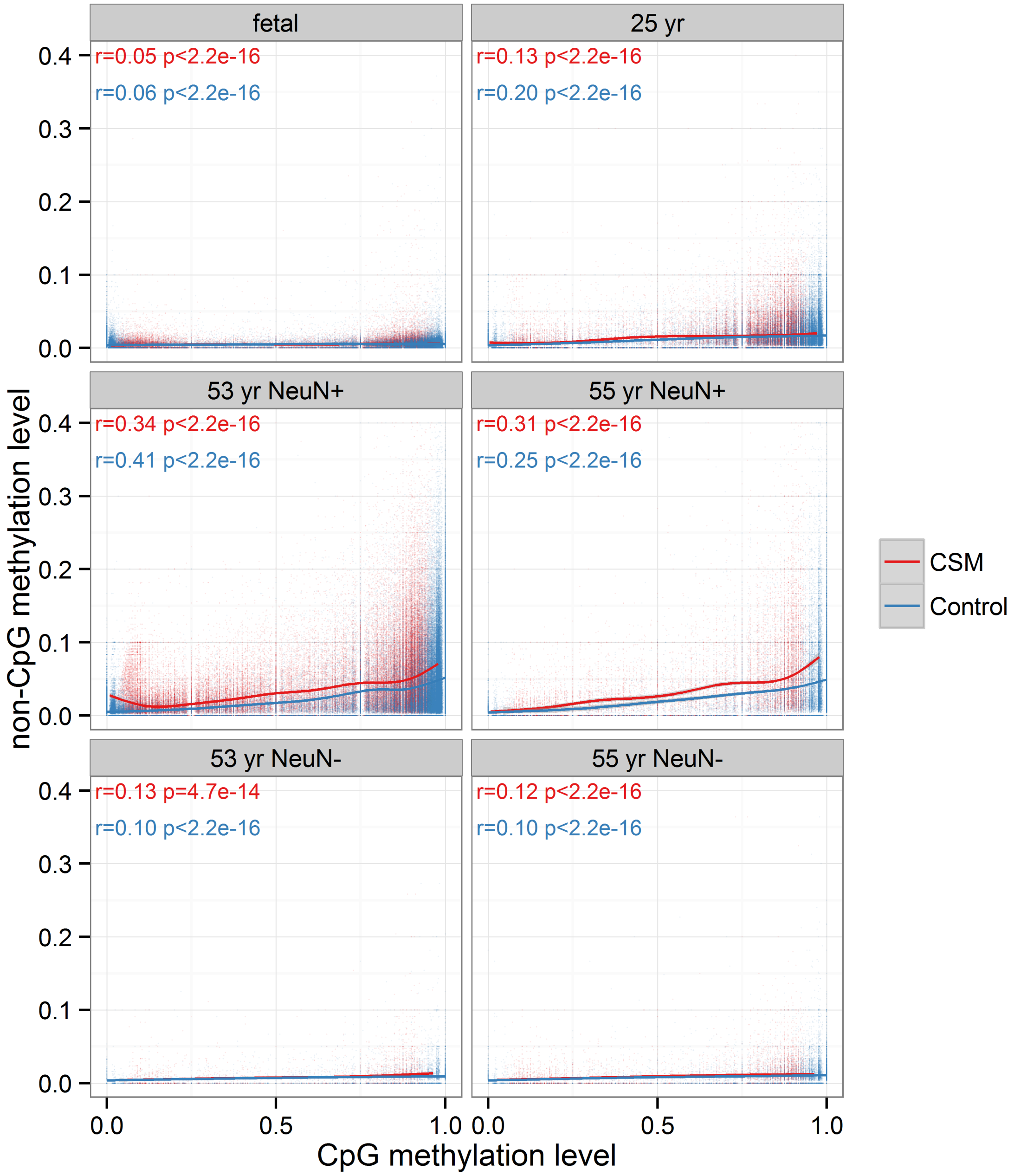

Supplementary Figure 5 (Xie)

Correlation between CpG and non-CpG methylation levels within pCSM and control regions

The x-axis and y-axis show the mC levels in CpG and non-CpG context for each 4-CpG segment, respectively. The curves show the smoothed spline. Pearson's r and p-values are shown.

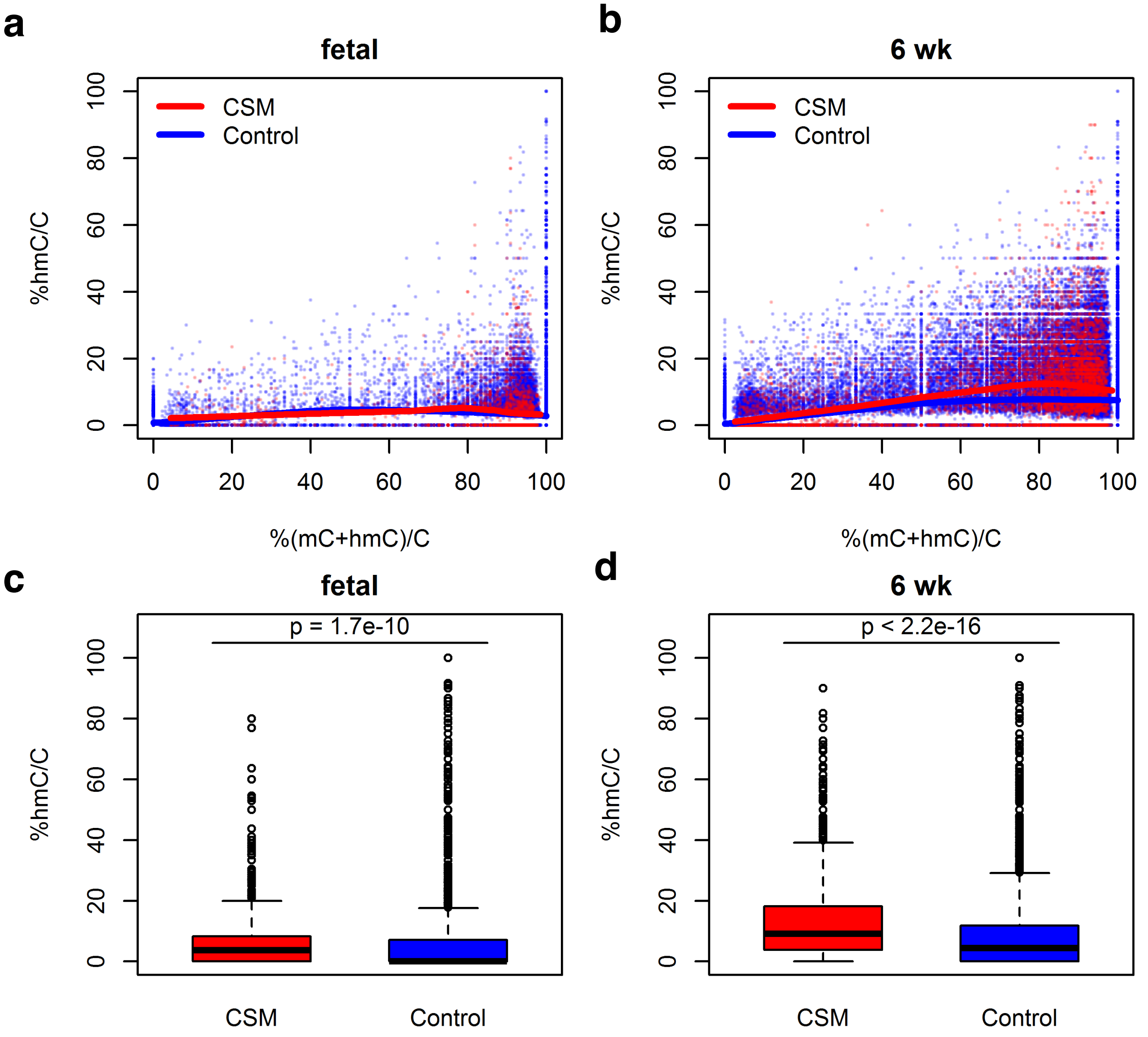

Supplementary Figure 6 (Xie)

5hmC occurrence in pCSM and control regions

(**a**,**b**) Scatter plots show the relationship between 5mC and 5hmC in CpG context within pCSM or control regions of fetal (**a**) and 6 wk (**b**) mouse brain methylomes, respectively. The lines are the smoothed curve computed using loess method. **(c,d**) Boxplots show the distributions of 5hmC level within pCSM or control regions of fetal (**c**) and 6 wk (**d**) mouse brain methylomes, respectively.

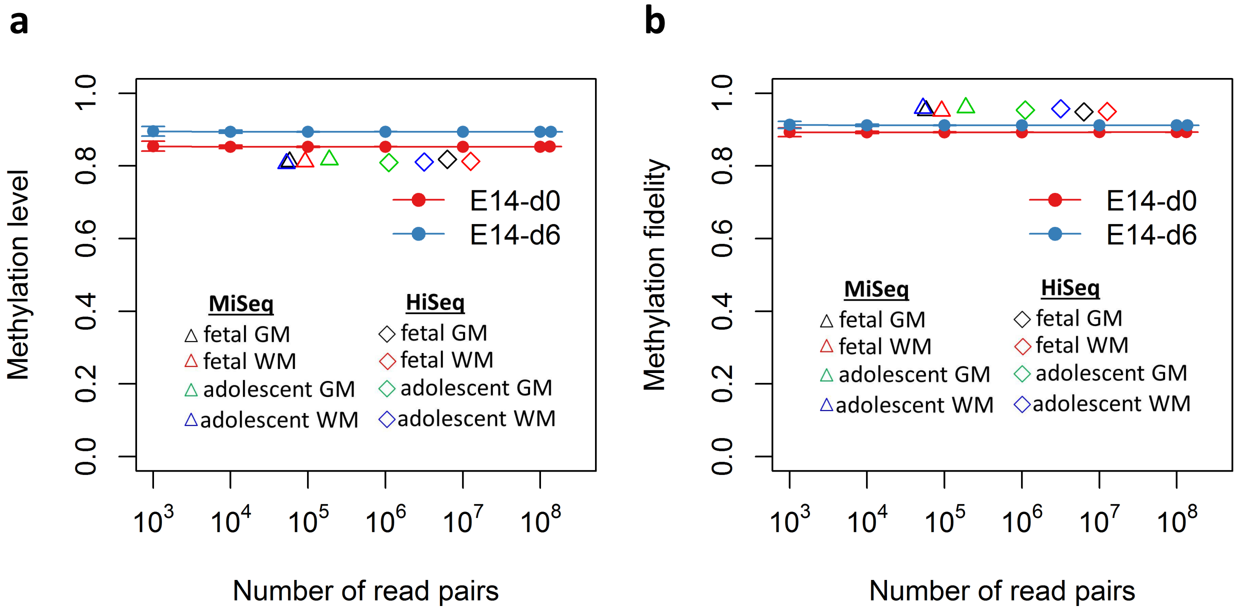

Supplementary Figure 7 (Xie)

Methylation level and fidelity estimated with hairpin bisulfite sequencing data at different sequencing depths

Various numbers of read pairs were selected randomly from the two previously published data sets generated for mouse embryonic stem cell E14 with genome-scale hairpin bisulfite sequencing technique[20]. The error bars show the standard deviation estimated from 100 times of simulations. The filled circles represent methylation level (**a**) or fidelity (**b**) determined with hairpin bisulfite sequencing data for mouse ESCs (E14-d0: mouse ESCs; E14-d6: mouse ESCs at day 6 with LIF withdrawn). In each line, the first six circles represent simulation results and the last circle shows the result using all reads generated. The open triangles and diamonds represent hairpin bisulfite sequencing data generated for human brain tissues in this study using MiSeq and HiSeq, respectively.
“fetal GM” represents human grey matter from a fetal frontal cortex.
“fetal WM” represents human white matter from a fetal frontal cortex.
“Adolescent GM” represents human grey matter from an adolescent frontal cortex.
“Adolescent WM” represents human white matter from an adolescent frontal cortex.

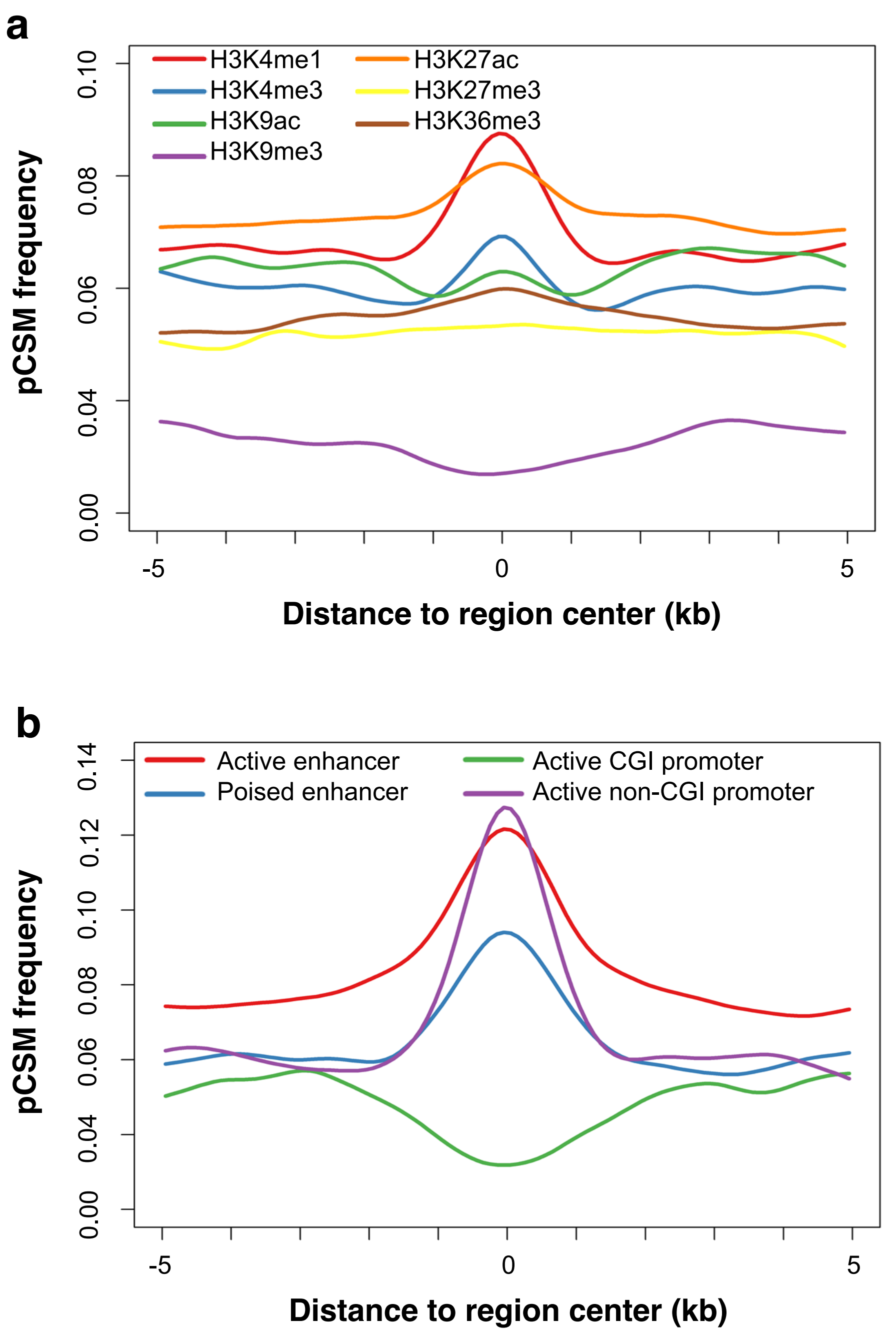

Supplementary Figure 8 (Xie)

Aggregate plots of pCSM segments surrounding different histone modifications (a) and regulatory elements (b) in adult human brain

The regulatory elements were annotated based on the combinations of histone modifications and the presence of CGI. Active promoters are with H3K4me3 mark, Active enhancers are with H3K27ac but without H3K4me3 mark, and poised enhancers are with H3K4me1 but without H3K27ac or H3K4me3.

Supplemental Table 1 Statistics for pCSM predicted from mammalian brain methylomes

Sample	Species	#mapped reads	#4CG segment (>=10X, noXY, noASM)	#predicted CSM segments	%pCSM frequency (unnormalized)	#pCSM segments after merging	#CpGs covered in pCSM segments	Total length of pCSM segments (bp)
Hs fetal	human	718,715,354	3,575,021	41,369	1.20%	26,960	124,217	1,260,254
Hs 35 do	human	754,934,101	1,014,430	25,030	2.50%	18,375	80,778	761,814
Hs 2 yr	human	769,797,789	1,255,212	47,202	3.80%	32,822	147,195	1,413,735
Hs 5 yr	human	749,639,389	1,105,550	40,181	3.60%	28,236	126,072	1,198,411
Hs 12 yr	human	757,884,985	1,133,642	46,538	4.10%	32,216	144,736	1,377,415
Hs 16 yr	human	746,971,503	1,289,687	57,503	4.50%	39,051	176,787	1,687,696
Hs 25 yr	human	725,663,402	1,133,157	50,024	4.40%	34,366	154,856	1,455,879
Hs 53 yrNeuN+	human	841,935,270	2,210,113	96,033	4.30%	61,121	284,305	2,891,375
Hs 53 yrNeuN-	human	476,665,175	217,362	5,636	2.60%	4,276	18,583	159,715
Hs 55 yrNeuN+	human	558,556,927	471,011	21,566	4.60%	15,516	68,659	619,658
Hs 55 yrNeuN-	human	539,415,159	362,534	9,925	2.70%	7,369	32,229	282,263
Mm fetal	mouse	553,383,395	162,533	5,218	3.20%	3,821	16,771	140,672
Mm 1 wk	mouse	385,489,360	235,332	8,455	3.60%	5,441	24,994	187,504
Mm 2 wk	mouse	618,425,417	1,439,393	55,568	3.90%	39,091	174,828	1,784,255
Mm 4 wk	mouse	672,968,248	1,197,542	57,001	4.80%	40,094	179,238	1,847,952
Mm 6 wk	mouse	667,130,394	805,885	40,088	5.00%	29,524	129,877	1,310,969
Mm 10 wk	mouse	448,962,696	797,566	23,794	3.00%	17,580	77,231	714,279
Mm 22 mo	mouse	682,097,075	944,270	51,795	5.50%	33,086	152,841	1,249,124
Mm 7 wkNeuN+	mouse	633,803,213	357,027	15,569	4.40%	9,864	45,567	383,268
Mm 7 wkNeuN-	mouse	676,970,294	587,176	20,815	3.50%	13,900	63,013	552,001
Mm 12 moNeuN+	mouse	436,577,319	194,533	9,765	5.00%	6,951	30,825	253,737
Mm 12 moNeuN-	mouse	332,614,011	102,534	3,343	3.30%	2,166	9,927	70,782

Supplementary Table 2 Pearson's correlations of pCSM statuses predicted for all 4-CpG segments in the methylomes of different samples.

a. human developmental stages (related to Figure 1E).

	fetal	35d	2yr	5yr	12yr	16yr	25yr
fetal		0.20	0.16	0.15	0.15	0.15	0.15
35d	0.20		0.23	0.21	0.21	0.20	0.20
2y	0.16	0.23		0.31	0.30	0.31	0.30
5y	0.15	0.21	0.31		0.31	0.31	0.30
12y	0.15	0.21	0.30	0.31		0.32	0.31
16y	0.15	0.20	0.31	0.31	0.32		0.33
25y	0.15	0.20	0.30	0.30	0.31	0.33	

b. mouse developmental stages (related to Supplemental Figure 2B).

	fetal	1wk	2wk	4wk	6wk	10wk	22mo
fetal		0.18	0.21	0.19	0.19	0.12	0.11
1wk	0.18		0.29	0.26	0.28	0.17	0.15
2wk	0.21	0.29		0.41	0.39	0.29	0.29
4wk	0.19	0.26	0.41		0.45	0.34	0.33
6wk	0.19	0.28	0.39	0.45		0.33	0.33
10wk	0.12	0.17	0.29	0.34	0.33		0.29
22mo	0.11	0.15	0.29	0.33	0.33	0.29	

c. human cell types (related to Supplemental Figure 3C)..

	53yr NeuN+	55yr NeuN+	53yr NeuN-	55yr NeuN-
53y NeuN+		0.37	0.16	0.19
55y NeuN+	0.37		0.15	0.19
53y NeuN-	0.16	0.15		0.30
55y NeuN-	0.19	0.19	0.30	

d. mouse cell types (related to Supplemental Figure 3D).

	7wk NeuN+	12mo NeuN+	7wk NeuN-	12mo NeuN-
7wk NeuN+		0.31	0.27	0.22
12mo NeuN+	0.31		0.24	0.18
7wk NeuN-	0.27	0.24		0.32
12mo NeuN-	0.22	0.18	0.32	

Supplemental Table 3a Summary of CSM predicted for human and mouse purified cells

Human (54,276 common 10X 4CG segment)		Mouse (36,692 common 10X 4CG segment)	
Sample	#pCSM segment	Sample	#pCSM segment
53yr NeuN+	4066	7wk NeuN+	3446
55yr NeuN+	3324	12mo NeuN+	3460
53yr NeuN-	1676	7wk NeuN-	3517
55yr NeuN-	1934	12mo NeuN-	2143

Supplemental Table 3b Number of shared pCSM segments between different samples

Human				
	53y_ NeuN+	55y_ NeuN+	53y_NeuN−	55y_NeuN−
53y_ NeuN+		1229	455	516
55y_ NeuN+	37%		413	516
53y_NeuN−	27%	25%		520
55y_NeuN−	27%	27%	31%	

Mouse				
	7wk_ NeuN+	12mo_ NeuN+	7wk_NeuN−	12mo_NeuN−
7wk_ NeuN+		1365	1145	840
12mo_ NeuN+	40%		1177	880
7wk_NeuN−	33%	34%		1045
12mo_NeuN−	39%	41%	49%	

Supplemental Table 4 Statistics of the hairpin-bisulfite sequencing data

Sample	Age	Tissue/cell type	Uniquely mapped read pairs (M)	Bisulfite conversion rate (%)	Genome covered (%)	CpG covered (%)	Genome sequencing depth for genome covered (X)	Average methylation level (%)	Average methylation fidelity (%)
fetal CG	22 weeks gestation	Cortical grey matter	6.4	99.8	8.2	9.7	10	81.8	94.9
fetal CW	22 weeks gestation	Cortical white matter	12.7	99.8	15	17.7	10.9	81.3	95
17y CG	17 years	Cortical grey matter	1.3	99.8	2.6	3.1	6.5	81.1	95.6
17y CW	17years	Cortical white matter	3.2	99.7	4.7	5.2	8.9	81.1	95.8

Supplemental Table 5a GO analysis for pCSM associated genes in human brain methylomes. Bonferroni adjusted p-values are indicated.

Category	Term	fetal	35d	2y	5y	12y	16y	25y	53y NeuN+	55y NeuN+	53y NeuN-	55y NeuN-
GOTERM_BP_FAT	GO:0030182~neuron differentiation	1	0.0036677	1.275E-07	3.039E-08	6.123E-09	1.43E-08	2.66E-11	1.169E-07	0.0001446	1	0.060787
GOTERM_BP_FAT	GO:0007155~cell adhesion	0.9997984	0.8186079	0.0812529	0.0007689	0.0010689	0.9848573	0.6720187	1	0.0400148	9.342E-10	2.877E-05
GOTERM_BP_FAT	GO:0022610~biological adhesion	0.9998879	0.8394253	0.0925335	0.0008995	0.0012577	0.9909776	0.7161087	1	0.0400148	9.342E-10	3.164E-05
GOTERM_BP_FAT	GO:0006357~regulation of transcription from RNA polymerase II prom	0.9124949	6.304E-05	1.288E-05	8.046E-06	1.754E-09	2.799E-08	4.915E-07	0.0002278	0.0006677	0.4668758	0.0129033
GOTERM_BP_FAT	GO:0048598~embryonic morphogenesis	0.9850514	0.2277992	0.0026876	0.5040555	2.027E-09	0.5260547	0.0396274	0.0343033	1	1	0.4421137
GOTERM_BP_FAT	GO:0007156~homophilic cell adhesion	0.0420162	1.528E-05	1	0.9999996	0.2910428	1	1	1	0.0054551	4.999E-09	7.579E-08
GOTERM_BP_FAT	GO:0048562~embryonic organ morphogenesis	0.393794	0.0348051	0.6792397	0.3767965	1.626E-08	0.9956556	0.1263553	0.637947	1	1	0.9861875
GOTERM_BP_FAT	GO:0016337~cell-cell adhesion	0.0675333	0.0026481	1	0.9800751	0.9959995	1	1	1	0.6232243	5.16E-08	6.539E-06
GOTERM_BP_FAT	GO:0031328~positive regulation of cellular biosynthetic process	1	3.105E-05	0.0010148	0.0038185	2.18E-06	1.373E-07	2.965E-05	0.0010603	0.0048711	1	0.3150969
GOTERM_BP_FAT	GO:0051173~positive regulation of nitrogen compound metabolic pro	1	8.427E-06	0.0037722	0.0033017	1.328E-05	2.37E-07	5.287E-05	0.0078498	0.017502	1	0.2731805
GOTERM_BP_FAT	GO:0009891~positive regulation of biosynthetic process	1	4.766E-05	0.0013601	0.0069093	4.715E-06	2.491E-07	4.492E-05	0.0006411	0.0039894	1	0.4232493
GOTERM_BP_FAT	GO:0010557~positive regulation of macromolecule biosynthetic proce	1	0.0001691	0.0006792	0.0092184	1.311E-06	3.422E-07	0.000295	0.0005388	0.0020028	1	0.6068586
GOTERM_BP_FAT	GO:0045941~positive regulation of transcription	1	0.000108	0.0048246	0.0471593	3.707E-06	5.476E-07	0.0010103	0.0050861	0.0011408	1	0.0813309
GOTERM_BP_FAT	GO:0032989~cellular component morphogenesis	1	0.002398	7.157E-06	0.0207527	6.148E-07	0.0664364	0.0006333	0.0289852	3.117E-05	1	0.0023916
GOTERM_BP_FAT	GO:0045935~positive regulation of nucleobase, nucleoside, nucleotide	1	5.526E-06	0.0107298	0.015573	4.578E-06	7.083E-07	0.0004688	0.0036697	0.0074018	1	0.2273625
GOTERM_BP_FAT	GO:0006355~regulation of transcription, DNA-dependent	1	0.0281162	0.0181186	6.728E-05	8.879E-06	8.057E-07	0.0018516	6.223E-06	0.512889	0.994076	0.8986776
GOTERM_BP_FAT	GO:0048568~embryonic organ development	0.9768438	0.0013864	0.1165066	0.0850355	9.114E-07	0.8353447	0.1953241	0.8031977	1	1	0.967088
GOTERM_BP_FAT	GO:0051252~regulation of RNA metabolic process	0.9999999	0.0870789	0.0156471	4.099E-05	9.551E-06	1.004E-06	0.0059639	1.275E-05	0.5949361	0.9999513	0.9238443
GOTERM_BP_FAT	GO:0010628~positive regulation of gene expression	1	0.0001039	0.0051876	0.0349483	6.648E-06	1.06E-06	0.000823	0.0046244	0.0025009	1	0.0489216
GOTERM_BP_FAT	GO:0051254~positive regulation of RNA metabolic process	0.9999999	0.0013103	0.0009712	0.0037449	1.163E-06	1.72E-05	0.0009212	0.0019197	0.0195212	1	0.0130118
GOTERM_BP_FAT	GO:0000902~cell morphogenesis	1	0.0011051	3.361E-06	0.0035744	1.912E-06	0.0081853	0.0001301	0.1689073	2.54E-06	1	0.0009008
GOTERM_BP_FAT	GO:0030030~cell projection organization	1	0.1412685	1.98E-06	0.020916	3.724E-05	3.754E-05	9.258E-06	0.0972415	0.0003074	1	7.792E-05
GOTERM_BP_FAT	GO:0007389~pattern specification process	1	0.1119283	0.3664456	0.0612651	2.018E-06	0.8031247	0.0806329	0.0210188	1	0.9987856	0.2914763
GOTERM_BP_FAT	GO:0048666~neuron development	1	0.3447523	6.856E-05	6.985E-05	0.0005595	2.518E-06	7.332E-06	0.0055932	0.0088786	1	0.592635
GOTERM_BP_FAT	GO:0045893~positive regulation of transcription, DNA-dependent	1	0.0007143	0.0007236	0.0079801	2.965E-06	1.088E-05	0.0006565	0.0017173	0.0120234	1	0.0316497
GOTERM_BP_FAT	GO:0031175~neuron projection development	1	0.270487	3.963E-06	0.0178968	0.0092879	3.005E-06	1.763E-05	0.0546033	0.0022102	1	0.0035483
GOTERM_BP_FAT	GO:0009792~embryonic development ending in birth or egg hatching	0.9431726	0.0068006	0.0228652	0.0084627	6.401E-06	0.0608187	0.008248	0.0192803	1	1	1
GOTERM_BP_FAT	GO:0032990~cell part morphogenesis	1	0.0158739	3.802E-05	0.3958316	7.98E-06	0.00042	0.0002365	0.6930356	0.0018244	1	0.0006925
GOTERM_BP_FAT	GO:0043009~chordate embryonic development	0.9875621	0.0041518	0.0348373	0.0079814	1.172E-05	0.0518925	0.0071256	0.0227999	1	1	1
GOTERM_BP_FAT	GO:0010604~positive regulation of macromolecule metabolic process	1	0.0004642	0.0020301	0.0191615	1.373E-05	0.0001162	0.0014235	0.001186	0.0949795	1	0.3700899
GOTERM_BP_FAT	GO:0048858~cell projection morphogenesis	1	0.0077137	2.209E-05	0.1498174	1.433E-05	0.0003784	0.0002589	0.3170567	0.0001916	1	0.0003329
GOTERM_BP_FAT	GO:0003002~regionalization	1	0.1383381	0.3870852	0.7372303	1.673E-05	0.9958082	0.0958279	0.0836558	1	1	0.7631403
GOTERM_BP_FAT	GO:0045944~positive regulation of transcription from RNA polymerase	0.9999997	0.0015145	0.0157775	0.0988647	2.104E-05	0.0003919	0.0001708	0.0271345	0.0745376	1	0.0611083
GOTERM_BP_FAT	GO:0048812~neuron projection morphogenesis	1	0.0094192	3.682E-05	0.0158102	5.004E-05	4.495E-05	2.45E-05	0.1406624	0.0014168	1	0.0004729
GOTERM_BP_FAT	GO:0030900~forebrain development	0.45084	0.0006692	0.004344	0.0080361	0.0076963	0.0028751	3.452E-05	0.1100362	0.0628511	1	0.99363
GOTERM_BP_FAT	GO:0043583~ear development	1	1	1	0.226407	3.502E-05	0.9978062	1	1	0.9999997	1	1
GOTERM_BP_FAT	GO:0000904~cell morphogenesis involved in differentiation	1	0.0014675	0.0007053	0.016276	5.018E-05	0.0005558	0.0008018	0.7493128	6.601E-05	1	0.0104973
GOTERM_BP_FAT	GO:0048667~cell morphogenesis involved in neuron differentiation	1	0.0028318	0.0004709	0.0092752	7.152E-05	0.0001059	6.112E-05	0.1293615	0.0009123	1	0.0060571
GOTERM_BP_FAT	GO:0045892~negative regulation of transcription, DNA-dependent	1	0.9618636	0.000901	0.1532992	0.0011344	6.242E-05	0.0554934	0.0237951	0.3380816	0.9664842	0.9991469
GOTERM_BP_FAT	GO:0051253~negative regulation of RNA metabolic process	1	0.9826968	0.0006401	0.1638059	0.0022135	0.0001835	0.1105472	0.0466873	0.3943581	0.9907377	0.9983447
GOTERM_BP_FAT	GO:0051056~regulation of small GTPase mediated signal transduction	0.0039388	0.1593699	0.0002599	0.0001978	0.0004686	0.0088165	0.0037471	1	1	1	0.3000853
GOTERM_BP_FAT	GO:0030036~actin cytoskeleton organization	1	0.999988	0.0007046	0.0272152	0.0021955	0.4117907	0.0374146	0.0002543	0.2679673	1	0.0044855
GOTERM_BP_FAT	GO:0007409~axonogenesis	1	0.0105787	0.0007423	0.0831889	0.0014462	0.0010233	0.000826	0.6740768	0.0003837	1	0.0017517
GOTERM_BP_FAT	GO:0010629~negative regulation of gene expression	1	0.4385333	0.0067098	0.6845725	0.276782	0.0004583	0.1099751	0.0323333	0.7438788	0.9996809	1
GOTERM_BP_FAT	GO:0016481~negative regulation of transcription	1	0.7261672	0.025783	0.5096418	0.464579	0.0006105	0.1191012	0.2286955	0.7986851	0.926267	1
GOTERM_BP_FAT	GO:0000122~negative regulation of transcription from RNA polymeras	1	1	0.0226355	0.6364154	0.0021303	0.0006285	0.2746231	0.2502721	0.493022	0.9375027	0.0460836
GOTERM_BP_FAT	GO:0007242~intracellular signaling cascade	1	1	0.0010759	0.0791309	0.0011154	0.0006667	0.6576564	0.4735669	1	1	1
GOTERM_BP_FAT	GO:0030029~actin filament-based process	1	0.9984019	0.0070776	0.359975	0.0017123	0.5351242	0.2532745	0.0010154	0.1662995	1	0.0321719
GOTERM_BP_FAT	GO:0048839~inner ear development	1	1	0.9988209	0.2009321	0.0012982	0.9999972	0.9999947	1	0.9996561	1	1
GOTERM_BP_FAT	GO:0045449~regulation of transcription	0.9954955	0.053711	0.0036592	0.0188114	0.0295269	0.0018711	0.0014992	0.0016394	0.5299515	0.989526	0.8871387
GOTERM_BP_FAT	GO:0035023~regulation of Rho protein signal transduction	0.134168	1	0.207915	0.0342787	0.9966799	0.3328175	0.0021384	1	1	1	1
GOTERM_BP_FAT	GO:0006928~cell motion	1	0.0128733	0.6085425	0.8422541	0.0024865	0.2245883	0.1434791	0.9999842	0.2552705	1	1
GOTERM_BP_FAT	GO:0045934~negative regulation of nucleobase, nucleoside, nucleotid	1	0.9808715	0.0110599	0.9857409	0.4630764	0.0025392	0.6570194	0.2517292	0.9766682	0.9859018	1
GOTERM_BP_FAT	GO:0051172~negative regulation of nitrogen compound metabolic pro	1	0.9506976	0.0068279	0.997458	0.3882466	0.0027665	0.4493475	0.3711673	0.996049	0.9574688	1
GOTERM_BP_FAT	GO:0045165~cell fate commitment	1	0.0157774	0.0905094	0.999885	0.0030069	0.3798131	0.1363772	0.0247352	0.0172907	0.8489586	0.8314905
GOTERM_BP_FAT	GO:0007010~cytoskeleton organization	1	1	0.0059809	0.037914	0.0076127	1	0.0163602	0.1234886	0.661477	1	0.0298368
GOTERM_BP_FAT	GO:0046578~regulation of Ras protein signal transduction	0.0332891	0.9437096	0.0461636	0.0060016	0.7035663	0.8265551	0.1458505	1	1	1	0.9999859
GOTERM_BP_FAT	GO:0031327~negative regulation of cellular biosynthetic process	1	0.8810297	0.0063361	0.9971199	0.0345728	0.033416	0.2772973	0.745081	0.997844	0.9976448	1
GOTERM_BP_FAT	GO:0007423~sensory organ development	1	1	1	0.8365648	0.00933	1	0.9937401	1	1	1	1
GOTERM_MF_FAT	GO:0043565~sequence-specific DNA binding	0.0922547	0.0010694	0.0001699	3.977E-09	4.304E-11	2.276E-14	2.814E-11	2.993E-11	0.9273744	0.3517491	0.1067167
GOTERM_MF_FAT	GO:0003700~transcription factor activity	0.0116095	1.625E-05	0.0003877	2.073E-10	9.663E-10	9.442E-10	2.785E-10	5.461E-10	0.6098781	0.4187548	0.000328
GOTERM_MF_FAT	GO:0008092~cytoskeletal protein binding	0.9999982	0.0004146	8.184E-06	0.0154405	7.008E-09	0.9887954	4.335E-07	0.1479633	0.0295778	1	0.0713456
GOTERM_MF_FAT	GO:0030528~transcription regulator activity	0.2853304	6.317E-05	5.366E-05	3.541E-06	2.499E-08	3.032E-06	3.817E-06	2.286E-05	0.2198935	0.4419809	0.4959275
GOTERM_MF_FAT	GO:0003779~actin binding	0.9999375	0.0223807	0.0001021	0.913165	1.481E-05	0.9727756	7.607E-06	0.2534254	0.0476692	1	0.9517746
GOTERM_MF_FAT	GO:0005509~calcium ion binding	0.9999999	0.2991891	0.1059315	0.0885649	0.5072222	0.17591	0.5491804	0.6523579	4.193E-05	0.2297968	0.9969595
GOTERM_MF_FAT	GO:0030695~GTPase regulator activity	0.0030045	0.0011947	9.666E-05	0.0007282	0.0098694	0.0106076	0.0163989	1	0.9999999	0.9934288	0.081512
GOTERM_MF_FAT	GO:0005085~guanyl-nucleotide exchange factor activity	0.0068583	0.3782166	0.0025745	0.0003073	0.0710239	0.1542619	0.042852	1	0.0408609	0.9996473	0.0001956
GOTERM_MF_FAT	GO:0005083~small GTPase regulator activity	0.0700624	0.5728441	0.0040086	0.0002139	0.7077258	0.5539691	0.7523813	1	1	1	0.3966791
GOTERM_MF_FAT	GO:0060589~nucleoside-triphosphatase regulator activity	0.0051607	0.0008383	0.0004272	0.0014664	0.0271945	0.0135654	0.0142677	1	1	0.9903431	0.1542956
GOTERM_MF_FAT	GO:0005088~Ras guanyl-nucleotide exchange factor activity	0.0072851	0.9068548	0.09469	0.0006648	0.6359002	0.2430295	0.3984837	1	0.9994701	1	0.0068724
GOTERM_MF_FAT	GO:0046872~metal ion binding	1	1	1	0.9979087	0.9830921	0.5662922	1	0.0041171	0.736402	1	0.9999997
GOTERM_MF_FAT	GO:0051020~GTPase binding	0.9984742	1	0.0043571	0.0485966	0.9991473	1	0.9706804	1	1	1	0.3350556
GOTERM_MF_FAT	GO:0043169~cation binding	1	1	1	0.9999979	0.9936912	0.8868424	1	0.0052319	0.7774967	1	0.9999999
GOTERM_MF_FAT	GO:0005089~Rho guanyl-nucleotide exchange factor activity	0.0108503	1	0.7152788	0.0057193	0.9698425	0.845939	0.126195	1	1	1	0.1573473
GOTERM_MF_FAT	GO:0003677~DNA binding	0.6127007	0.9948541	0.941214	0.3609668	0.9183146	0.0488906	0.0816668	0.0087495	1	1	0.7205044
GOTERM_MF_FAT	GO:0017016~Ras GTPase binding	0.9999999	1	0.0091152	0.3657198	1	1	0.9999991	1	1	1	0.999983
GOTERM_MF_FAT	GO:0031267~small GTPase binding	1	1	0.0099318	0.0225764	0.9993983	1	0.9987681	1	1	1	0.6657156
GOTERM_CC_FAT	GO:0044459~plasma membrane part	0.4077772	0.0416583	0.0004808	0.0015174	1.164E-05	6.416E-06	1.402E-09	0.0001362	0.1082669	0.943267	0.9942624
GOTERM_CC_FAT	GO:0042995~cell projection	0.9985052	0.9478561	0.0250375	0.0004579	2.041E-08	0.0013711	7.366E-05	0.0086116	0.4736946	1	0.0071263
GOTERM_CC_FAT	GO:0005886~plasma membrane	0.9999996	0.0018118	0.0012271	5.016E-05	0.0005763	0.0038388	1.096E-05	1	0.0130668	0.0019337	0.01696
GOTERM_CC_FAT	GO:0031252~cell leading edge	1	1	0.0126726	0.0134199	1.105E-05	0.270394	0.0003317	1	0.9999368	0.9230352	0.010321
GOTERM_CC_FAT	GO:0015629~actin cytoskeleton	0.9740264	0.0001132	0.0031507	0.0125888	1.153E-05	0.9993433	0.0661029	0.1999065	0.9918859	0.9997183	0.6107294
GOTERM_CC_FAT	GO:0070161~anchoring junction	0.9999991	1	0.6264282	0.2322705	1.609E-05	1	0.0158057	0.9999845	1	0.9999999	0.3297681
GOTERM_CC_FAT	GO:0005912~adherens junction	0.999994	0.9999157	0.0573145	0.4323689	5.563E-05	1	0.0005968	0.9854954	1	0.9976074	0.70459
GOTERM_CC_FAT	GO:0043005~neuron projection	1	1	0.9945377	0.0061052	0.0001056	0.0004757	0.0001614	0.0343531	0.5596759	1	1
GOTERM_CC_FAT	GO:0005856~cytoskeleton	0.5591326	0.0002113	0.0484865	0.1267743	0.0042821	1	0.0104257	0.0167565	1	1	0.6379805
GOTERM_CC_FAT	GO:0031226~intrinsic to plasma membrane	0.9999958	0.9999155	0.8618339	1	0.9999463	0.0043719	0.0093435	0.9906911	0.99939	1	1
GOTERM_CC_FAT	GO:0001726~ruffle	1	1	0.0496085	0.0844979	0.0065423	0.995862	0.1639725	1	0.9437868	0.9999999	0.0239152
GOTERM_CC_FAT	GO:0030054~cell junction	0.1608239	1	0.432586	0.2290396	0.2839479	1	0.0087324	0.1477398	1	1	0.9999851
GOTERM_CC_FAT	GO:0005887~integral to plasma membrane	1	0.9999678	0.8595172	1	0.9999999	0.0091303	0.0313073	0.9985869	0.996976	1	1

Supplemental Table 5b GO analysis for pCSM associated genes in mouse brain methylomes. Bonferroni adjusted p-values are indicated.

Category	Term	fetal	1wk	2wk	4wk	6wk	10wk	22mo	7wk NeuN+	12mo NeuN+	7wk NeuN-	12mo NeuN-
GOTERM_BP_FAT	GO:0006357~regulation of transcription from RNA polymerase II promoter	1	1	0.0000136	3.51E-12	0.00000191	0.037308342	2.77E-09	0.034696112	1	1	0.696873879
GOTERM_BP_FAT	GO:0051252~regulation of RNA metabolic process	1	1	0.048236883	6.47E-09	0.664571403	0.013719602	0.0000288	0.995710483	1	0.99999995	0.999639998
GOTERM_BP_FAT	GO:0006355~regulation of transcription, DNA-dependent	1	1	0.029247049	2.74E-08	0.614006673	0.018358163	0.0000188	0.993497738	1	0.999999487	0.996256163
GOTERM_BP_FAT	GO:0045944~positive regulation of transcription from RNA polymerase II promoter	1	1	0.00000982	0.000000154	0.002344251	0.37165561	0.012735107	0.999291609	1	1	1
GOTERM_BP_FAT	GO:0030030~cell projection organization	1	1	0.00016	0.000000185	0.004404082	0.00000344	0.003116381	0.000188	1	0.898376125	1
GOTERM_BP_FAT	GO:0031328~positive regulation of cellular biosynthetic process	1	1	0.00000574	0.000000229	0.115387296	0.999964989	0.040304285	1	1	1	0.999995977
GOTERM_BP_FAT	GO:0010628~positive regulation of gene expression	1	1	0.0000255	0.000000275	0.070528825	0.769343047	0.010469776	1	1	1	1
GOTERM_BP_FAT	GO:0048568~embryonic organ development	1	1	0.0000375	0.001625355	0.010107438	0.00178811	0.000000377	1	1	0.999870551	1
GOTERM_BP_FAT	GO:0045941~positive regulation of transcription	1	1	0.00001	0.000000407	0.129378783	0.97381298	0.024925415	1	1	1	1
GOTERM_BP_FAT	GO:0048598~embryonic morphogenesis	1	1	0.000000526	0.00000728	0.152348013	0.006112656	0.0000268	1	1	0.878661692	1
GOTERM_BP_FAT	GO:0048562~embryonic organ morphogenesis	1	1	0.051964538	0.000735	0.966267823	0.039481784	0.000000644	1	1	1	1
GOTERM_BP_FAT	GO:0030182~neuron differentiation	1	1	0.0000952	0.00000088	0.000355	0.000000684	0.0000406	0.024203537	1	0.998490597	1
GOTERM_BP_FAT	GO:0045165~cell fate commitment	1	1	0.008758198	0.00000434	0.999999999	0.000853	0.000000791	1	1	1	1
GOTERM_BP_FAT	GO:0009891~positive regulation of biosynthetic process	1	1	0.0000104	0.000000938	0.143938026	0.999981152	0.083212767	1	1	1	0.999999214
GOTERM_BP_FAT	GO:0010557~positive regulation of macromolecule biosynthetic process	1	1	0.00000585	0.00000214	0.050312318	0.998095256	0.132335031	1	1	1	0.999999953
GOTERM_BP_FAT	GO:0035023~regulation of Rho protein signal transduction	1	1	0.0000495	0.00000253	0.000122	0.107864946	0.266499964	0.859348173	1	1	1
GOTERM_BP_FAT	GO:0051254~positive regulation of RNA metabolic process	1	1	0.0000578	0.00000387	0.023084431	0.554917137	0.02043549	1	1	1	1
GOTERM_BP_FAT	GO:0051173~positive regulation of nitrogen compound metabolic process	1	1	0.0000097	0.00000396	0.273138201	0.999999962	0.043283487	1	1	1	1
GOTERM_BP_FAT	GO:0030900~forebrain development	1	1	0.147845219	0.018665067	0.237436161	0.00000446	1	0.76077788	1	0.186199347	1
GOTERM_BP_FAT	GO:0051056~regulation of small GTPase mediated signal transduction	1	1	0.000133	0.00000478	0.00000862	0.0000789	0.001314954	0.778516617	0.565003611	0.736106339	1
GOTERM_BP_FAT	GO:0045449~regulation of transcription	1	1	0.278764289	0.00000566	1	0.999773266	0.035838044	1	1	1	0.99999995
GOTERM_BP_FAT	GO:0000902~cell morphogenesis	1	1	0.0000321	0.000033	0.001193952	0.00000705	0.000497	0.551727659	1	0.881918676	1
GOTERM_BP_FAT	GO:0045935~positive regulation of nucleobase, nucleoside, nucleotide and nucleic a	1	1	0.00000949	0.00000794	0.206026572	0.99864411	0.036213967	1	1	1	1
GOTERM_BP_FAT	GO:0031175~neuron projection development	1	1	0.007972939	0.018786024	0.358805593	0.00000837	0.051712465	0.007884484	1	0.935837505	1
GOTERM_BP_FAT	GO:0045893~positive regulation of transcription, DNA-dependent	1	1	0.0000492	0.0000101	0.037744965	0.596681506	0.019223088	0.999999996	1	1	1
GOTERM_BP_FAT	GO:0051253~negative regulation of RNA metabolic process	1	1	1	0.001216635	0.020857813	0.512618209	0.0000162	0.999998459	1	1	1
GOTERM_BP_FAT	GO:0010604~positive regulation of macromolecule metabolic process	1	1	0.0000165	0.000374	0.025528844	0.959208026	0.430798141	1	1	1	1
GOTERM_BP_FAT	GO:0046578~regulation of Ras protein signal transduction	1	1	0.00084	0.0000186	0.000287	0.01534605	0.188803027	0.502592207	0.813878291	0.99998952	1
GOTERM_BP_FAT	GO:0045892~negative regulation of transcription, DNA-dependent	1	1	1	0.002278307	0.014143061	0.603439168	0.0000186	0.999999996	1	1	1
GOTERM_BP_FAT	GO:0032989~cellular component morphogenesis	1	1	0.000239	0.000151	0.030917564	0.000019	0.000378	0.551011083	1	0.999910366	1
GOTERM_BP_FAT	GO:0042127~regulation of cell proliferation	1	1	0.0000267	0.001679887	0.255274663	0.099229814	0.003743757	1	1	1	1
GOTERM_BP_FAT	GO:0001501~skeletal system development	1	1	0.87233541	0.001037303	0.278522675	0.996620228	0.0000309	1	1	0.978205952	1
GOTERM_BP_FAT	GO:0051172~negative regulation of nitrogen compound metabolic process	1	1	1	0.066667482	0.920378552	0.998901696	0.0000385	0.999999747	1	1	1
GOTERM_BP_FAT	GO:0006928~cell motion	1	0.999995762	0.000117	0.0000396	0.001247334	0.020196035	0.945351781	0.84143151	1	0.999999918	1
GOTERM_BP_FAT	GO:0045934~negative regulation of nucleobase, nucleoside, nucleotide and nucleic	1	1	1	0.043574843	0.860388435	0.999994694	0.0000441	0.999998628	1	1	1
GOTERM_BP_FAT	GO:0048666~neuron development	1	1	0.000731	0.000528	0.001254032	0.0000504	0.010894794	0.018071117	1	0.999984383	1
GOTERM_BP_FAT	GO:0030036~actin cytoskeleton organization	0.923810535	1	0.0000545	0.796970112	0.964445014	0.086399619	0.006899333	1	1	0.999999992	1
GOTERM_BP_FAT	GO:0007242~intracellular signaling cascade	1	1	0.217783067	0.000109	0.028400763	0.222630342	0.0000712	0.999997078	1	1	1
GOTERM_BP_FAT	GO:0031327~negative regulation of cellular biosynthetic process	1	1	1	0.023176648	0.185075931	0.999954877	0.0000754	1	1	1	1
GOTERM_BP_FAT	GO:0007423~sensory organ development	1	1	0.324368472	0.0000921	0.278842631	0.000245	0.000246	0.944935985	1	0.851821664	1
GOTERM_BP_FAT	GO:0016481~negative regulation of transcription	1	1	1	0.029873631	0.733232926	0.999719045	0.0000936	1	1	1	1
GOTERM_BP_FAT	GO:0022610~biological adhesion	1	0.01977393	0.178568078	0.002306977	0.000108	0.718601044	0.005273613	1	0.674459703	0.086546064	1
GOTERM_BP_FAT	GO:0007155~cell adhesion	1	0.01977393	0.178568078	0.002306977	0.000108	0.718601044	0.005273613	1	0.674459703	0.086546064	1
GOTERM_BP_FAT	GO:0000122~negative regulation of transcription from RNA polymerase II promote	1	1	1	0.006504417	0.033351836	0.999969215	0.00014	0.993791793	1	1	1
GOTERM_BP_FAT	GO:0030029~actin filament-based process	0.990065637	1	0.000151	0.997040181	0.999999475	0.330865059	0.022301481	0.999999977	1	0.999999999	1
GOTERM_BP_FAT	GO:0009890~negative regulation of biosynthetic process	1	1	1	0.008502389	0.30134378	0.999999261	0.000184	1	1	1	1
GOTERM_BP_FAT	GO:0048663~neuron fate commitment	1	1	1	0.000259	1	0.999924202	0.46264028	1	1	1	1
GOTERM_BP_FAT	GO:0001525~angiogenesis	1	1	0.000266	0.287664902	0.006885868	0.999963184	0.04311551	1	1	1	1
GOTERM_BP_FAT	GO:0048858~cell projection morphogenesis	1	1	0.014569058	0.000314	0.345289609	0.000838	0.338899039	0.044587237	1	0.953882066	1
GOTERM_BP_FAT	GO:0048514~blood vessel morphogenesis	1	1	0.001377758	0.000321	0.015844114	0.060849716	0.018195845	1	1	1	1
GOTERM_BP_FAT	GO:0007389~pattern specification process	1	1	0.001412299	0.000369	0.051581171	0.004352864	0.000661	0.99985417	1	1	1
GOTERM_BP_FAT	GO:0010558~negative regulation of macromolecule biosynthetic process	1	1	1	0.013260211	0.743933595	1	0.000369	1	1	1	1
GOTERM_BP_FAT	GO:0003002~regionalization	1	1	0.013598722	0.000374	0.906294822	0.084857722	0.008041965	1	1	0.995667097	1
GOTERM_BP_FAT	GO:0000904~cell morphogenesis involved in differentiation	1	1	0.001501342	0.018903175	0.178945058	0.000413	0.020834093	0.034855487	1	0.998026994	1
GOTERM_BP_FAT	GO:0051270~regulation of cell motion	1	1	0.999702504	0.000491	0.057448561	0.999783199	0.814341364	1	1	1	1
GOTERM_BP_FAT	GO:0030334~regulation of cell migration	1	1	0.909236739	0.000647	0.251216822	0.99999161	0.605601158	1	1	1	1
GOTERM_BP_FAT	GO:0010629~negative regulation of gene expression	1	1	1	0.023193032	0.996662002	0.999660686	0.000676	1	1	1	1
GOTERM_BP_FAT	GO:0032990~cell part morphogenesis	1	1	0.123587773	0.000747	0.812739688	0.008024946	0.700007936	0.030517983	1	0.999941754	1
GOTERM_BP_FAT	GO:0001944~vasculature development	1	1	0.005756961	0.000806	0.045879505	0.108191622	0.002679011	1	1	1	1
GOTERM_BP_FAT	GO:0048812~neuron projection morphogenesis	1	1	0.019711908	0.00413498	0.647485777	0.000869	0.218429797	0.028934439	1	0.577283678	1
GOTERM_BP_FAT	GO:0001568~blood vessel development	1	1	0.003894117	0.001625355	0.024019855	0.075615307	0.004033902	1	1	1	1
GOTERM_BP_FAT	GO:0043009~chordate embryonic development	1	1	0.165854629	0.002096926	0.011914566	0.002660583	0.004066212	1	1	1	1
GOTERM_BP_FAT	GO:0009792~embryonic development ending in birth or egg hatching	1	1	0.180277356	0.00261243	0.007106496	0.002341785	0.00436209	1	1	1	1
GOTERM_BP_FAT	GO:0045596~negative regulation of cell differentiation	1	1	0.999999332	0.006443987	0.999999252	0.002729037	0.002427494	1	1	1	1
GOTERM_BP_FAT	GO:0007507~heart development	1	1	0.307429934	0.178014279	0.687728816	0.002955726	0.003083284	1	1	1	1
GOTERM_BP_FAT	GO:0048667~cell morphogenesis involved in neuron differentiation	1	1	0.042553121	0.027708364	0.984363022	0.004943093	0.743372606	0.027934793	1	0.900730936	1
GOTERM_BP_FAT	GO:0016477~cell migration	1	1	0.005065786	0.008874813	0.063059712	0.33094361	1	1	1	1	1
GOTERM_BP_FAT	GO:0007409~axonogenesis	1	1	0.121052386	0.038567948	0.992858714	0.005427338	0.821169217	0.018668343	1	0.549568323	1
GOTERM_BP_FAT	GO:0035239~tube morphogenesis	1	1	0.050394267	0.006242896	0.793805318	0.968802496	0.890339253	1	1	1	1
GOTERM_BP_FAT	GO:0008284~positive regulation of cell proliferation	1	1	0.006360105	0.10749073	1	0.998281638	0.201370686	1	1	0.999988044	1
GOTERM_BP_FAT	GO:0051094~positive regulation of developmental process	1	1	0.797252839	0.006743632	0.908741672	0.511980058	0.186690066	1	1	1	1
GOTERM_BP_FAT	GO:0035295~tube development	1	1	0.038023532	0.009932245	0.667558932	0.24541716	0.074194945	1	1	1	1
GOTERM_MF_FAT	GO:0003700~transcription factor activity	1	1	9.3E-09	3.93E-19	0.000373	0.00000197	2.61E-10	0.032969685	1	0.015968783	0.72262565
GOTERM_MF_FAT	GO:0043565~sequence-specific DNA binding	1	1	0.0000296	1.68E-17	0.0000431	0.005789326	3.07E-09	0.001009538	1	0.174009424	0.863169326
GOTERM_MF_FAT	GO:0030528~transcription regulator activity	1	1	0.00000555	3.58E-11	0.071488467	0.00268037	0.000000006	0.827480722	1	0.870625758	0.876275852
GOTERM_MF_FAT	GO:0005088~Ras guanyl-nucleotide exchange factor activity	1	0.999967234	0.000273	1.29E-08	0.0000169	0.088839981	0.136293561	0.998680462	1	0.712139986	1
GOTERM_MF_FAT	GO:0005085~guanyl-nucleotide exchange factor activity	1	1	0.011550714	3.48E-08	0.0000186	0.505519518	0.713595018	0.976261482	1	0.298457454	1
GOTERM_MF_FAT	GO:0005089~Rho guanyl-nucleotide exchange factor activity	1	0.999961757	0.00023	0.000000217	0.000206	0.604802524	0.279679488	0.945700557	1	0.999947498	1
GOTERM_MF_FAT	GO:0003677~DNA binding	1	0.97744243	0.020774993	0.000000521	1	0.975943883	0.053545091	0.999932583	1	0.999999981	0.999721298
GOTERM_MF_FAT	GO:0005083~small GTPase regulator activity	1	1	0.0000019	0.000465	0.000000884	0.114862475	0.003772389	0.508703432	0.795213259	0.426871811	1
GOTERM_MF_FAT	GO:0030695~GTPase regulator activity	1	1	0.00000766	0.00000212	0.00000179	0.006724852	0.003566366	0.589750113	0.020030788	0.053843243	1
GOTERM_MF_FAT	GO:0060589~nucleoside-triphosphatase regulator activity	1	1	0.0000402	0.00000204	0.00000384	0.016107644	0.011874106	0.742828363	0.032862083	0.100711557	1
GOTERM_MF_FAT	GO:0003779~actin binding	0.308731052	1	0.0000101	0.001285184	0.122208142	0.015289033	0.001890721	0.253951361	0.992704981	0.685824926	1
GOTERM_MF_FAT	GO:0008092~cytoskeletal protein binding	0.841841718	0.999999645	0.000442	0.052360872	0.996225725	0.02166182	0.000048	0.292931693	1	0.601161909	1
GOTERM_MF_FAT	GO:0003705~RNA polymerase II transcription factor activity, enhancer binding	1	1	0.999462464	0.001623462	0.99994252	1	1	0.999617102	1	1	1
GOTERM_CC_FAT	GO:0005886~plasma membrane	1	0.00212765	0.00000329	3.07E-11	1.48E-10	2.85E-12	3E-13	0.922687626	0.0000124	0.0000115	0.138114642
GOTERM_CC_FAT	GO:0005856~cytoskeleton	0.242340397	0.954994499	0.0000142	0.000471	0.0000938	1.45E-09	0.00000734	0.004607314	0.149719376	0.0000495	0.998613251
GOTERM_CC_FAT	GO:0044459~plasma membrane part	0.999991398	0.994617255	0.037174044	0.000306	0.011657423	0.000000195	9.74E-08	0.8639455	0.002220879	0.097946032	0.276763494
GOTERM_CC_FAT	GO:0030054~cell junction	1	0.037269542	0.04094723	0.095421013	0.001353388	0.001284927	0.005846598	0.238365755	0.0000602	0.015631062	1
GOTERM_CC_FAT	GO:0044430~cytoskeletal part	0.923290292	1	0.253732102	0.248436636	0.379811678	0.000121	0.227096807	0.981672156	1	0.185683011	1
GOTERM_CC_FAT	GO:0042995~cell projection	0.958490535	1	0.018720569	0.009391296	0.002445547	0.000727	0.000874	0.999999779	1	0.042376392	0.999999738
GOTERM_CC_FAT	GO:0015629~actin cytoskeleton	0.041718372	1	0.001286796	0.021117228	0.293518709	0.13084368	0.154865236	0.984644705	0.999958764	0.990498316	0.962434394
GOTERM_CC_FAT	GO:0070161~anchoring junction	1	0.758044899	0.995599235	0.003931189	0.074776629	0.076851657	0.244683158	1	0.787310444	0.999980425	1
GOTERM_CC_FAT	GO:0031252~cell leading edge	1	0.775221993	0.160792693	0.00924403	0.13865452	0.446565194	0.064153556	0.004651471	0.800450856	0.999999886	1

Supplemental Table 6 Summary of "omics" data used in this study

GEO accession	Organism	Tissue/cell type	Age	Data type	Brief description	Reference
GSE67482	Human	brain cortex	fetal (22wk of gestation) and 17y	hairpin bisulfite-Seq	Hairpin bisulfite-Seq data for human brain	This study
GSE47966	Human<br>Mouse	brain frontal cortex, neuron, glia	fetal, adolescent and adult	methylC-Seq	Methylomes for several developmental stages and two cell types (neuron and glia) of human and mouse brain	Lister et al, Science 2013[1]
GSE29184	Mouse	brain cortex	8wk	ChIP-Seq	Chromatin maps for H3K4me1, H3K4me3 and H3K27ac	Shen et al, Nature 2014[2]
GSE16368	Human	brain	75y/81y	ChIP-Seq	Chromatin maps for H3K4me1, H3K4me3, H3K9ac, H3K9me3, H3K27ac, H3K27me3 and H3K36me3	Bernstein et al, Nat Biotechnol 2010[3]
NHGRI GWAS catalog	Human	Various	Various	GWAS	NHGRI GWAS catalog	Welter et al, NAR 2014[4]

Reference:

1. Lister R, Mukamel EA, Nery JR, Urich M et al. Global epigenomic reconfiguration during mammalian brain development. Science 2013 Aug 9;341(6146):1237905. PMID: 23828890
2. Shen Y, Yue F, McCleary DF, Ye Z et al. A map of the cis-regulatory sequences in the mouse genome. Nature 2012 Aug 2;488(7409):116-20. PMID: 22763441
3. Bernstein BE, Stamatoyannopoulos JA, Costello JF, Ren B et al. The NIH Roadmap Epigenomics Mapping Consortium. Nat Biotechnol 2010 Oct;28(10):1045-8. PMID: 20944595
4. Welter D, MacArthur J, Morales J, Burdett T, et al. The NHGRI GWAS Catalog, a curated resource of SNP-trait associations. Nucleic Acids Research, 2014, Vol. 42: D1001-D1006.
